# Advancing precision photothermal therapy by integrating armored gold nanostars with real-time photoacoustic thermometry and imaging

**DOI:** 10.1126/sciadv.adx6350

**Published:** 2025-08-13

**Authors:** Aidan J. Canning, Tri Vu, Luca Menozzi, Paul Klippel, Xinrong Chen, Jianing Chen, Theresa Charity, Khang Hoang, Joseph J. Yang, Yun Jing, Gregory M. Palmer, Junjie Yao, Tuan Vo-Dinh

**Affiliations:** ^1^ Department of Biomedical Engineering, Duke University, Durham, NC 27708 USA.; ^2^ Fitzpatrick Institute for Photonics, Duke University, Durham, NC 27708, USA.; ^3^ Stephenson School of Biomedical Engineering, University of Oklahoma, Norman, OK 73019, USA.; ^4^ Graduate Program in Acoustics, Penn State University, University Park, PA 16802, USA.; ^5^ Department of Radiation Oncology, Duke University School of Medicine, Durham, NC 27710, USA.; ^6^ Department of Surgery, Duke University School of Medicine, Durham, NC 27710, USA.; ^7^ Department of Neurology, Duke University School of Medicine, Durham, NC 27710, USA.; ^8^ Department of Chemistry, Duke University, Durham, NC 27708 USA.

## Abstract

Nanoparticle-mediated photothermal therapy (PTT) is a promising strategy for cancer treatment; however, nanoparticle instability and lack of precise imaging tools for real-time temperature monitoring during therapy and nanoparticle tracking have hindered investigations in animal models. To address these critical issues, we present a theranostic platform that seamlessly integrates armored core–gold nanostar (AC-GNS)–mediated PTT with full-view photoacoustic computed tomography (PACT), enabling nanoparticle tracking and real-time imaging-guided PTT in deep tissues. The AC-GNS platform delivered exceptional photostability and thermal resilience beyond those of conventional nanoparticles while serving as a high-performance contrast agent for PACT and a photothermal transducer for PTT. Integrating AC-GNS–mediated PTT with noninvasive PACT enabled whole-body nanoparticle tracking, PTT treatment monitoring via thermal imaging, and thermal dose determination, culminating in a 100% survival rate in a murine bladder cancer model without long-term treatment-related toxicity. This theranostic platform lays the foundation for broader research applications and provides opportunities for advancing solid tumor treatment and response assessment research.

## Teaser

Theranostic nanoparticles and noninvasive temperature monitoring enable the refinement of light-based cancer therapy.

## INTRODUCTION

Light-mediated cancer therapies have serious potential owing to their precision and minimal invasiveness (*1*). However, substantial clinical obstacles remain in realizing localized therapeutic efficacy, minimizing off-target damage, and providing real-time monitoring of treatment response. Nanoparticle-mediated photothermal therapy (PTT) has emerged as a promising solution to these challenges and has recently been successful in treating prostate cancer in humans (*2*–*4*). In nanoparticle-mediated PTT, thin-walled or high-aspect-ratio nanoparticles with a morphology optimized to increase near-infrared (NIR) optical absorption accumulate in the tumor microenvironment (TME), thereby increasing the rate of photothermal conversion relative to the surrounding tissues. This increase in photothermal conversion substantially increases the temperature within the TME, selectively causing tumor cell death, antitumor immune activation, and vascular disruption (*4*–*6*). Gold nanoparticles (GNPs) are particularly attractive photothermal transducers owing to their tunable morphology-dependent optical properties and high biocompatibility (*7*, *8*). However, the clinical translation of nanoparticle-mediated PTT has been substantially hampered by two major limitations: the inherent instability of high-aspect-ratio GNPs under laser irradiation, which can compromise their photothermal conversion efficiency and imaging capabilities, and the lack of robust deep tissue imaging tools for real-time nanoparticle tracking and temperature monitoring during therapy (*1*, *9*, *10*). These challenges impede accurate treatment characterization and the ability to optimize therapeutic outcomes. Therefore, more accurate treatment characterization methods are needed to accelerate the translation of novel PTT schemes for treating solid tumors.

To overcome these critical limitations, we present a theranostic platform that integrates highly stable armored core–gold nanostar (AC-GNS) nanoparticles with full-view photoacoustic computed tomography (PACT), enabling real-time imaging-guided PTT with simultaneous deep tissue thermometry. PACT is well-suited for nanoparticle tracking and thermal mapping because of its intrinsic sensitivity to optical absorption and temperature (*11*). By solely relying on changes in NIR optical absorption caused by nanoparticle accumulation, deep-tissue, label-free, nonionizing detection of GNPs with high contrast and sensitivity has been achieved using PACT (*12*, *13*). Furthermore, by leveraging the temperature-dependent Gruineisen parameter, PACT is inherently sensitive to temperature changes, offering the potential for real-time thermal monitoring during PTT (*14*, *15*). In contrast to magnetic resonance thermometry, which is often used when performing PTT in human subjects, PACT is a cost-effective alternative with superior temporal resolution and accessibility to novel PTT schemes in animal models (*16*, *17*). Traditional PTT monitoring techniques, such as thermal cameras and invasive temperature probes, only provide superficial or local temperature measurements (*3*, *15*, *17*).

To date, integrating the PACT and GNP-mediated PTT methods has been challenging. High-aspect-ratio GNPs, such as nanorods and nanostars, can undergo spherical conversion when exposed to high-energy pulsed laser energies used for PACT or at biological temperatures for extended periods (*10*, *18*, *19*). The optical absorption spectra of these particles shift markedly due to this change, which undercuts the photothermal transduction performance and leads to inaccurate quantification of nanoparticle concentration and thermal changes using PACT (*20*, *21*). A range of nanoparticle stabilization strategies have emerged in attempts to curb this deformation and retain high-aspect-ratio nanoparticle features. These strategies include soft stabilization methods with surface-adsorbed proteins, polymers, and thiolated compounds (*22*–*24*), rigid silica layers as protective shells (*25*–*27*), or incorporating metals with high melting temperatures in the nanoparticle design (*28*). However, soft encapsulation methods have relatively low thermal surface stability (*29*), limiting their ability to maintain high-aspect-ratio morphologies. Rigid protective layers often come at the expense of more challenging surface functionalization, in vivo particle stability, and overall particle size, which all can negatively affect nanoparticle accumulation within the TME via the enhanced permeation and retention (EPR) effect or prevent intravenous administration entirely (*25*, *30*–*32*). In terms of nanoparticle composition selection, gold is associated with the lowest cytotoxicity (*7*, *8*). For gold nanoparticle platform selection, the synthesis of gold nanorods often involves cytotoxic ionic surfactants, such as cetyltrimethylammonium bromide, making surfactant-free high-aspect ratio platforms such as GNSs a more biocompatible and translational alternative (*10*). Therefore, there is a need to improve the photo- and thermal stability of surfactant-free high-aspect-ratio plasmonic GNPs while preserving particle size and solution stability to maintain in vivo performance and leverage the EPR effect for tumor targeting.

In response to the critical need for high-aspect-ratio plasmonic nanoparticles with improved photo and thermal stability and robust deep-tissue monitoring during photothermal therapy, we developed an AC-GNS nanoparticle platform. This novel nanoarchitecture featuring a GNS within a hollow gold shell facilitates superior photo and thermal stability, resulting in constant nanoparticle optical properties throughout the treatment. Stable nanoparticle optical properties ensure accurate and highly sensitive detection via PACT imaging, excellent and reliable photothermal transduction performance during PTT, and accurate noninvasive PACT thermometry. A murine bladder cancer flank-tumor model was used to demonstrate the integration of AC-GNS–mediated PTT and PACT for nanoparticle tracking and thermometry. Whole-body PACT imaging provided high-resolution deep-tissue AC-GNS biodistribution and pharmacokinetics for nanoparticle dosages from 2.5 to 0.5 pM administered via retro-orbital injection. Noninvasive real-time thermal mapping and thermal dose calculations were performed during PTT using the same low-particle dosages. Long-term survival studies after PTT revealed that 100% of animals that received AC-GNS–mediated PTT remained completely tumor-free after 6 months, with no signs of long-term treatment-related damage or toxicity. Beyond this specific murine bladder cancer application, the highly stable AC-GNS design principles and integrated PACT/PTT methodology offer a broadly adaptable platform for advancing nanoparticle-based theranostics and accelerating PTT innovations and translations.

## RESULTS

### AC-GNS synthesis and characterization

**Fig. 1.**
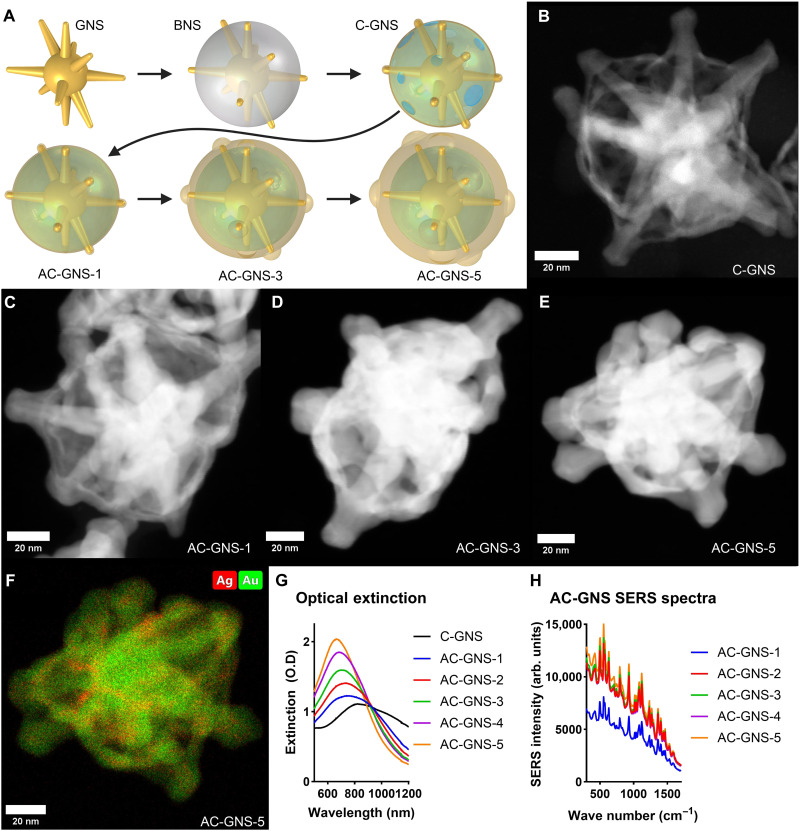
AC-GNS nanoparticle synthesis and characterization. (**A**) Scheme depicting the development of the AC-GNS morphology. (**B**) Representative STEM image of unsealed C-GNS particle. (**C**) Representative STEM image of AC-GNS-1 particle. (**D**) Representative STEM image of AC-GNS-3 particle. (**E**) Representative STEM image of AC-GNS-5 particle. (**F**) EDS-STEM image depicting the elemental makeup of an AC-GNS particle. (**G**) Extinction spectra of different AC-GNS morphologies. (**H**) Average SERS spectra of different AC-GNS spectra, *N* = 5. O.D, optical density.

To synthesize AC-GNS particles, a layer of silver is first deposited on the core GNS particle, resulting in bimetallic nanostars (BNSs). Particles are then centrifuged and dispersed in a basic solution containing polyvinyl pyrrolidone (PVP) to ensure particle stability and ascorbic acid to facilitate the reduction of gold onto the surface of the BNS particles in a galvanic replacement-free reaction to minimize gold-silver alloy formation (Fig. 1A) (*33*, *34*). Gold-coated BNS particles are then dispersed into an acidic hydrogen peroxide solution heated to 55°C, which removes the internal silver layer and encourages the formation of insoluble silver chloride for subsequent removal. This process results in the formation of caged GNSs (C-GNS) (Fig. 1B and fig. S1A). After several washing steps to remove all insoluble silver chloride, the particles were then concentrated and added to an ethanol solution containing 1,1′,3,3,3′,3′-hexamethylindotricarbocyanine iodide (HITC) dye. The now dye-loaded particles were then dispersed into a solution containing PVP and ascorbic acid, where varying amounts of gold chloride were added to encapsulate the dye within the hollow region of the particle and increase outer shell thickness, resulting in a range of morphologies referred to as AC-GNS-1 to AC-GNS-5 (Fig. 1, C to E, and fig. S1, B to D). After the final gold coating, the AC-GNS particles were washed several times in ethanol and then incubated in an ethanol solution containing SH-PEG_5000_-COOH to ensure the removal of all surface-bound PVP and dye and to maximize particle monodispersity and prevent opsonization in vivo (*35*). To confirm that the dye was encapsulated within the interior of the AC-GNS, the surface-enhanced Raman scattering (SERS) signal of particle aliquots with and without final gold coating was recorded before and after a 30-min exposure to a 6% H_2_O_2_ solution (fig. S1E). The aliquot that did not receive the final gold coating retained only 8.4% of its initial signal after H_2_O_2_ exposure, whereas the AC-GNS aliquot retained 62% of its original signal or nearly eight times more of the original signal compared to the unsealed counterpart, confirming that the dye is encapsulated within the particle (fig. S1F).

The nanoparticle tracking analysis results (fig. S1G) indicate that the average hydrodynamic diameter of the nanoparticles increased from 106 nm for AC-GNS-1 particles to 125 nm for AC-GNS-5 particles. Inductively coupled plasma mass spectrometry (ICP-MS) results (fig. S1H) reveal that the amount of gold per nanoparticle increases linearly [coefficient of determination (*R*^2^) = 0.989] from AC-GNS-1 to AC-GNS-5. The efficacy of the galvanic replacement-free preparation method can be evaluated using energy-dispersive x-ray spectroscopy (EDS)–scanning transmission electron microscopy (STEM) images of the representative AC-GNS-5 particles (Fig. 1F). The average gold and silver concentrations for AC-GNS-3, the nanoparticle morphology with an intermediate shell thickness, determined by ICP-MS was 64.7 mg of gold/liter with a relative standard deviation (RSD) of 0.3% and 6.7 mg of silver/liter with an RSD of 0.6%. This morphology was further characterized using dynamic light scattering, which yielded an average nanoparticle diameter of 98 ± 1.03 nm and a polydispersity index of 0.124 ± 0.012 (fig. S1I).

The AC-GNS morphology is a single continuous metallic gold structure; therefore, by increasing the thickness of the outer shell, the oscillation frequency of conduction electrons throughout the particle or the localized surface plasmon resonant (LSPR) frequency is altered, which is reflected in the nanoparticle extinction spectra. As the shell thickness increases, the extinction spectra blue shift (Fig. 1G), a trend consistent with other works examining gold thin-shelled structures (*36*). To observe the effects of shell thickness on SERS enhancement, spectra from each of the five formulations were recorded (Fig. 1H). The AC-GNS-1 formulation had the lowest signal, ~50% of the maximum generated by AC-GNS-5. The remaining three formulations tested had SERS intensities within 10% of AC-GNS-5.

### Plasmonic investigation of AC-GNS structure

**Fig. 2.**
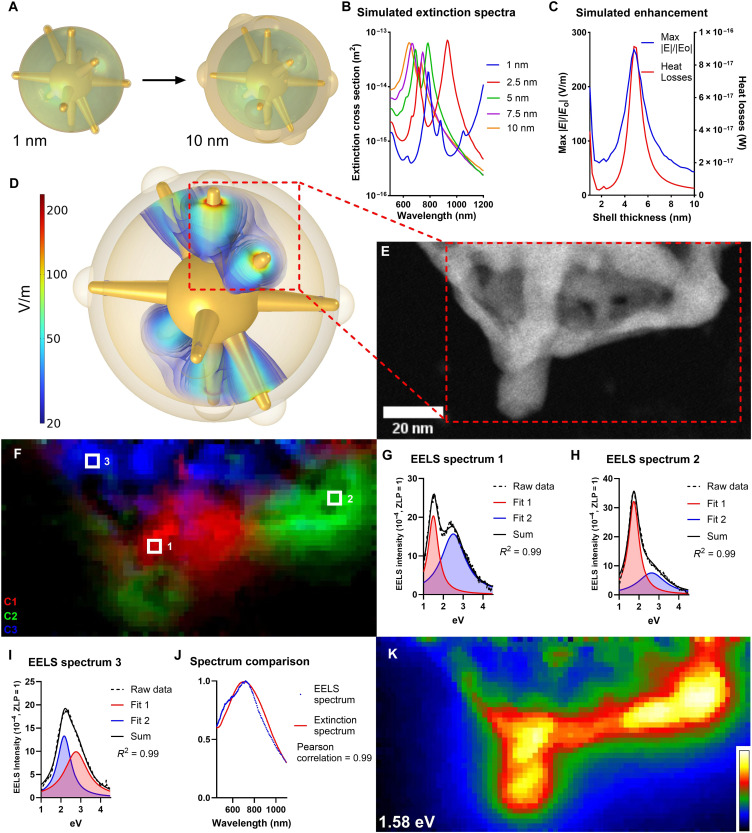
Investigation of AC-GNS plasmonic properties. (**A**) Scheme depicting the range of shell thicknesses simulated in COMSOL. (**B**) Simulated extinction cross section of AC-GNS models as a function of outer shell thickness. (**C**) Simulated maximum electric field enhancement and heat losses generated by AC-GNS models as a function of outer shell thickness at 785 nm. (**D**) Electric field enhancement generated by the top-performing AC-GNS model. (**E**) High-angle annular dark-field–STEM image of particle analyzed via EELS. (**F**) Distribution of some spatially trackable spectral components generated from EELS analysis. (**G**) EELS spectrum recorded from location 1. (**H**) EELS spectrum recorded from location 2. (**I**) EELS spectrum recorded from location 3. (**J**) Comparison between optical extinction spectrum of AC-GNS solution and EELS spectrum of entire spectrum image. (**K**) EELS mapping depicting LDOS centered at 1.58 eV.

COMSOL Multiphysics was used to model the emergent plasmonic properties of nanoparticle models derived from the observed AC-GNS morphologies. For these simulations, the internal GNS structure was maintained at a constant configuration, and the outer shell thickness was varied from 1 to 10 nm (Fig. 2A). More information about the particle geometry, material properties, and simulation conditions can be found in Materials and Methods. The extinction coefficient between 500 and 1200 nm was calculated for several models to examine the effects of outer shell thickness on the nanoparticle extinction spectrum (Fig. 2B). In line with the experimental extinction spectrum of AC-GNS particles (Fig. 1G), there is a noticeable blue shift in spectral components as the thickness of the outer shell increases. To determine the optimal shell thickness for SERS enhancement, the local electric field generated by the nanoparticle model upon incident exposure to 785-nm light was simulated for shell thicknesses ranging from 1 to 10 nm in steps of 0.2 nm (Fig. 2C). Along with the maximum electric field enhancement value, the heat losses value was also solved for, which is derived by integrating the resistive losses (W/m^3^) with the volume of the nanoparticle, providing additional information about the spatial electric field enhancement around the nanoparticle model (*37*). The maximum electric field intensity and heat losses values occurred at an AC-GNS model shell thickness of 4.8 nm with values of 268.6 V/m and 9.14 × 10^−17^ W, respectively, and most closely resembles the synthesized AC-GNS-3 morphology (Fig. 1D). The area of greatest electric field enhancement for the top-performing model (Fig. 2D) occurs within the hollow region of the nanoparticle in a toroidal zone around the branch-shell junction and is made possible by the continuous nature of the AC-GNS morphology. The difference in trends seen between the experimental SERS data (Fig. 1H), where AC-GNS-5 produces a slightly higher SERS signal than AC-GNS-3, and simulated enhancement data (Fig. 2C), which suggest that AC-GNS-3 should be the top performing morphology, is caused by the change in the nanoparticle solution extinction spectrum as a function of outer shell thickness. As the outer shell increases in thickness beyond 5 nm, the SERS enhancement theoretically decreases but so does the NIR extinction of the nanoparticle solution, thereby increasing the chances of scattered light reaching the detector.

To directly interrogate the emergent plasmonic properties of the AC-GNS nanoarchitecture, we conducted a monochromatic electron energy loss spectroscopy (EELS) study, where the inelastic scattering of electrons is used to characterize the LSPR modes of nanoparticles (*38*). EELS was performed on a portion of an AC-GNS-3 particle (Fig. 2E) using a Titan 80-300 aberration-corrected STEM equipped with a Gatan EELS spectrometer. Following the best practices for EELS data analysis (*38*), the zero-loss peak of each collected spectrum was normalized to 1 to account for signal loss caused by the gold structure scattering electrons out of the EEL spectrometer aperture. To denoise the data and gain insight into the distribution of spatially trackable spectral components generated from EELS analysis, principal components analysis followed by a varimax rotation in the spatial domain was performed to ensure non-negativity of spectral loadings using the top eight ranked components in DigitalMicrograph software, some of which had distinct peaks at 1.59, 1.75, 2.26, and 2.37 eV (fig. S2A). The superposition (Fig. 2F) of the non-negative scores (fig. S2, B to D) for these representative spatially trackable spectral components indicate regions across the spectrum image that may contain distinct LSPR modes but not information about the modes themselves.

To obtain mode-specific information, the top eight ranked components were recombined, and the tail of the zero-loss peak was fit with a logarithmic function between 0.45 and 0.95 eV and subtracted as background (*38*). Spectra were analyzed from locations 1, 2, and 3 (Fig. 2F), corresponding to EELS spectra 1, 2, and 3 (Fig. 2, G to I). Because of the heterogeneity of the AC-GNS structure, we expected overlapping LSPR modes in each of the EELS spectra and fit the background-subtracted data with multiple Lorentzian curves (*38*, *39*). For all three spectra, a dual-Lorentzian model fit the raw data with an *R*^2^ value of 0.99, with curves centered at 1.50 and 2.50 eV for spectrum 1 (Fig. 2G), 1.74 and 2.66 eV for spectrum 2 (Fig. 2H), and 2.16 and 2.76 eV for spectrum 3 (Fig. 2I). Peaks at 1.50 and 1.74 eV are within the range of branch modes and peaks ranging from 2.16 to 2.76 eV are within the range of core modes previously reported for GNS (*40*, *41*). The sum of the three spectra, the sum of all Lorentzian fits, and the spectra integrated over the whole image are nearly identical (fig. S2E), demonstrating that the spectra selected for this analysis provided a comprehensive view of the particle as a whole. Furthermore, the EELS spectrum exhibits a Pearson’s correlation value of 0.99 with the optical extinction spectra of the AC-GNS-3 solution, indicating that the particles selected for EELS analysis are an accurate representative of the group (Fig. 2J) (*39*).

LSPR mapping was performed across the entire spectrum image to observe the local density of optical states (LDOS) as a function of energy (movie S1). Spatially resolved EELS revealed that there is a high LDOS as a result of multiple overlapping plasmon modes centered at 1.58 eV (Fig. 2K), corresponding to 785-nm excitation, in strong agreement with COMSOL simulations showing high electric field enhancement at the branch-shell junction at 785 nm (Fig. 2D). In addition, all NIR resonant modes are concentrated at branch-shell junctions and along the shell wall, suggesting that the shell of the AC-GNS morphology confers increased NIR activity broadly. Coupling this spatial-energy agreement with experimental SERS data showing high SERS enhancement at 785 nm (Fig. 1H) demonstrate that AC-GNS morphology can produce optically bright plasmon modes at internal branch-shell junctions.

### Enhanced AC-GNS stability

**Fig. 3.**
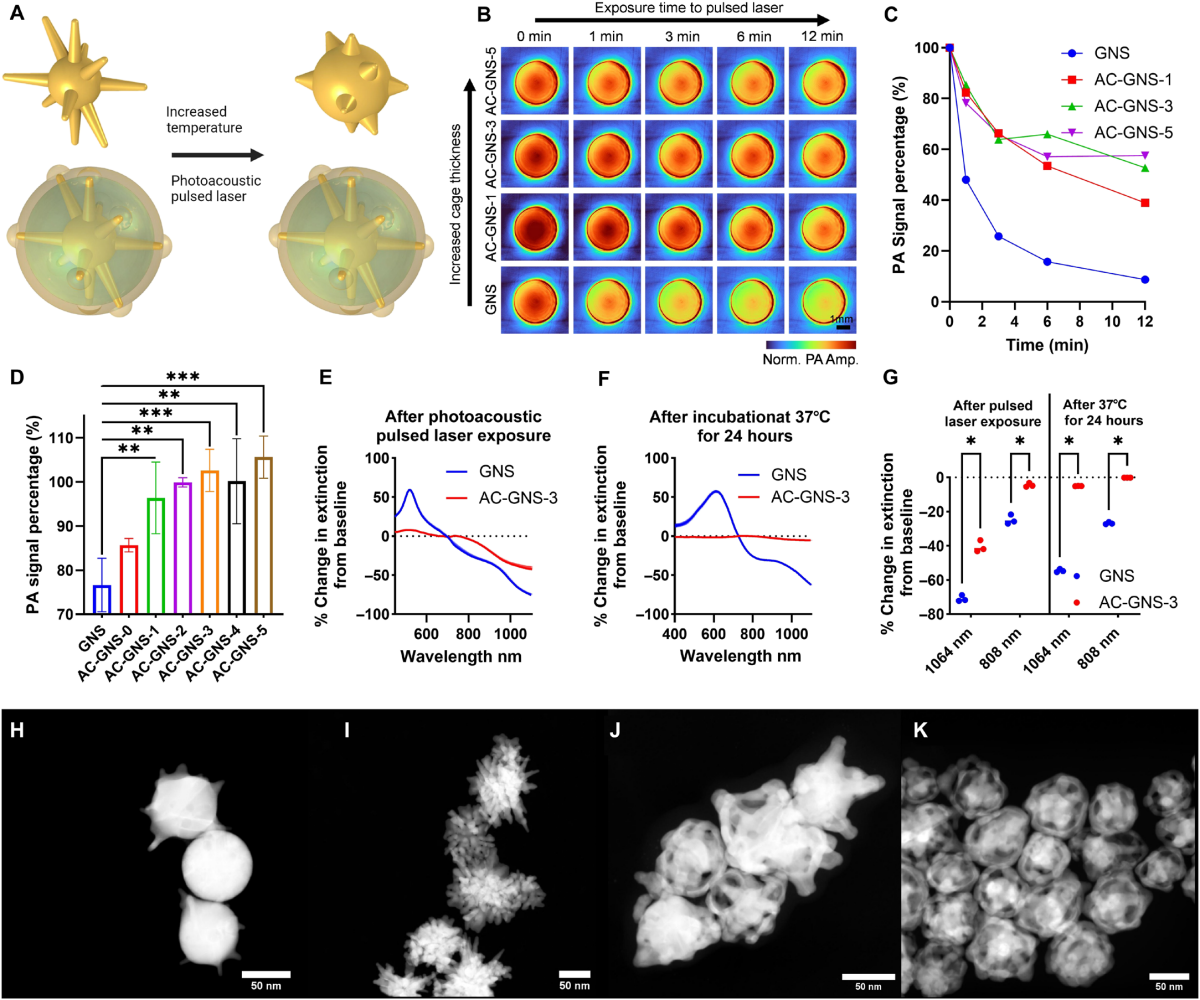
Enhanced stability of AC-GNS particles. (**A**) Scheme depicting proposed improved stability of AC-GNS particles. (**B**) Normalized PA amplitude of different nanoparticle formations after pulsed laser exposure at 110 mJ/cm^2^. (**C**) Quantified decrease in PA amplitude with pulsed laser exposure at 110 mJ/cm^2^. (**D**) Percentage of initial PA amplitude at 1064 nm after 12 min of pulsed laser exposure of 1.1 mJ/cm^2^. (**E**) Change in percent extinction for GNS and AC-GNS particles after 12 minutes of laser exposure at 110 mJ/cm^2^. (**F**) Change in percent extinction for GNS and AC-GNS particles after incubating for 24 hours at 37°C. (**G**) Quantification of percentage change in extinction for 808 and 1064 nm, *N* = 3. (**H**) STEM image of GNS particles after 12 min of exposure to 1064-nm laser at 110 mJ/cm^2^. (**I**) STEM image of GNS particles after 12 min of exposure to 1064-nm laser at 1.1 mJ/cm^2^. (**J**) STEM image of AC-GNS-3 particles after 12 min of exposure to 1064-nm laser at 110 mJ/cm^2^. (**K**) STEM image of AC-GNS-3 particles after 12 min of exposure to 1064-nm laser at 1.1 mJ/cm^2^. *P ≤ 0.05, **P ≤ 0.01, and ***P ≤ 0.001.

Photo and thermal stability testing was performed to determine whether the AC-GNS morphology could provide an advantage over traditional GNS (Fig. 3A). The initial photostability test was conducted by exposing nanoparticle aliquots contained within a liquid sample holder to pulsed 1064-nm laser illumination with a high optical fluence of 110 mJ/cm^2^ or 9.9 mJ [slightly higher than the ANSI safety limit of 100 mJ/cm^2^ for 1064 nm (*42*)] for 1, 3, 6, and 12 min (fig. S3, A and B). The decrease in the PA signal intensity for all AC-GNS morphologies at 1064 nm was substantially lower than that of the traditional GNS morphology (Fig. 3, B and C). The PA intensity of the GNS sample decreased by 91.3% after 12 min of exposure, whereas the intensities of the AC-GNS-1, AC-GNS-3, and AC-GNS-5 aliquots decreased by 61.1, 47.2, and 42.4%, respectively. To investigate AC-GNS stability using the full-view ring array PACT system, we repeated the pulsed laser photostability test using a low fluence of 1.1 mJ/cm^2^ and illumination scheme used for in vivo imaging studies performed in this work (fig. S4, A to C). This power density was estimated on the basis of Monte Carlo simulations of mouse PA imaging (fig. S5, A and B), detailed information on simulation parameters can be found in Materials and Methods. The simulation showed an illumination area of ~9 cm^2^ using only voxels >50% of maximum power. Therefore, the particles received roughly 9.9 mJ again for this test. After the in vivo mimicking photostability test, there was no measurable decrease in PA intensity for AC-GNS morphologies 2 to 5 (Fig. 3D). A one-way analysis of variance (ANOVA) with Dunnett’s test revealed that the PA intensity of GNS significantly decreased after 12 min of pulsed laser exposure compared to AC-GNS morphologies 1 to 5 (*P* < 0.01). On the basis of the nanoparticle extinction spectra (Fig. 1G) and photostability tests (Fig. 3, B to D), the AC-GNS-3 morphology was selected for further investigation because of its high NIR absorption and significantly improved stability.

**Fig. 4.**
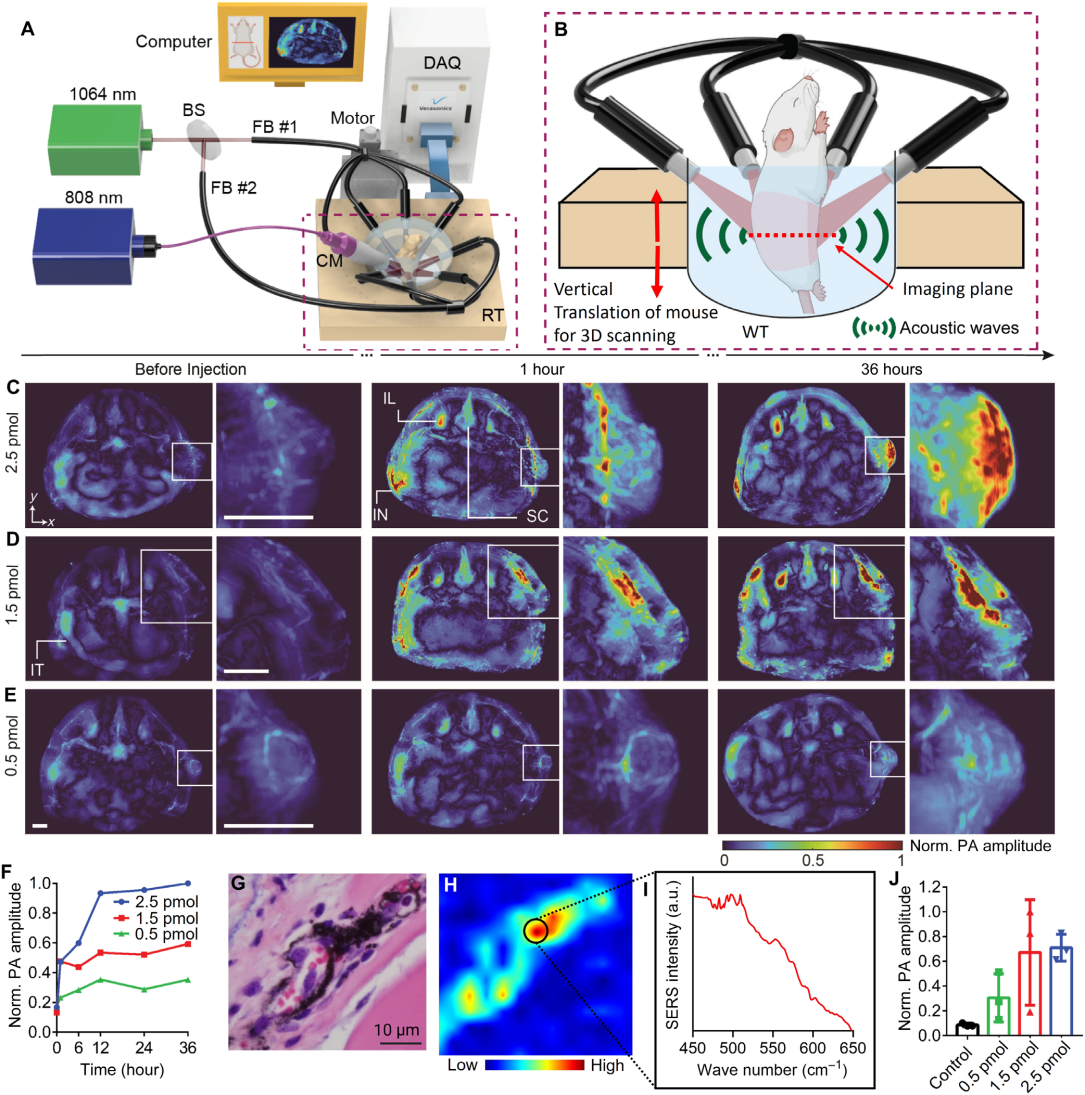
Deep tissue AC-GNS biodistribution imaging with full-view PACT system. (**A**) Schematics of the PACT/PTT system. (**B**) In vivo imaging setup for whole-body PACT scanning and PA thermometry during PTT. (**C** to **E**) Representative longitudinal PA maximum amplitude projection (MAP) at the tumor slices (~1-cm elevational range) with 0.5-, 1.5-, and 2.5-pmol AC-GNS dosages, respectively. (**F**) Average PA signal at the tumor over time showing following AC-GNS injection (**G**) Representative hematoxylin and eosin (H&E) histological image showing the AC-GNS accumulation in the tumor, as confirmed by the SERS hyperspectral imaging (**H**). (**I**) SERS spectra collected from region of highest intensity. (**J**) Average PA signal at the tumor for each dosage group. Scale bar, 2 mm. IL, iliac lymph node; IN, inguinal lymph node; SC, spinal cord; IT, intestine; DAQ, data-acquisition instrument; FB, fiber bundle; RT, ring-array transducer; CM, collimator; WT, water tank.

The percentage change in the optical extinction (Fig. 3E) of the GNS and AC-GNS particles before and after repeating the high-energy photostability test (Fig. 3, B and C) revealed a sharp increase in the optical extinction of the GNS solution at 520 nm, indicating the conversion of high-aspect-ratio particles to spheres (*18*). Thermal stability at 37 C° for 24 hours was assessed to mimic the accumulation conditions in vivo after intravenous injection. Again, there was a large percentage change from the baseline in the optical extinction of the GNS solution relative to that of the AC-GNS solution (Fig. 3F). An unpaired *t* test of the stability test results (Fig. 3G) revealed that AC-GNS particles had significantly greater thermal and photostability than traditional GNS particles at 808 and 1064 nm, which are the wavelengths for PTT and PACT used in this study, respectively. Photostability testing revealed that the extinction at 1064 and 808 nm decreased by 70.9 and 24.8%, on average, for the GNS sample and 40.6 and 4.5%, on average, for the AC-GNS sample. Thermal stability testing revealed that the extinction at 1064 and 808 nm decreased by 54.6 and 26.8%, on average, for the GNS sample and 5.1 and 0.1%, on average, for the AC-GNS sample. These results indicate that the AC-GNS morphology addresses the critical limitation of high-aspect-ratio nanoparticle susceptibility to photothermal and thermal degradation, thus enabling more reliable and repeatable outcomes in therapeutic and diagnostic applications. STEM imaging revealed that the GNS were nearly spherical after the high-energy test (Fig. 3H), whereas the branches of the GNS particles began to mesh together, forming a nano-waffle fry-like structure after the low-energy test (Fig. 3I). In contrast, only some outer shell breakdown was observed for the AC-GNS particles, and the original morphology was mostly conserved after the high-energy test. Furthermore, no differences in the morphology of the AC-GNS particles were observed after low-energy tests (Fig. 3K).

The spherical conversion of high-aspect-ratio nanoparticles results from the high surface energy characteristic of these structures (*18*, *43*). In the case of nanostars, the branches are pulled toward the center of the particle, decreasing in aspect ratio and subsequently surface energy until the particle becomes a sphere. In contrast, the AC-GNS morphology has a continuous gold structure on both ends of the high-aspect-ratio branches. This geometric anchoring of the branches creates a local surface energy minimum and provides improved structural stability. If the branches move toward either the particle shell or core, by necessity, then part of the branch will have to become even thinner, increasing in surface energy, which is unfavorable. In the case of the high-energy pulsed laser test, we do see some total branch and shell collapse evidenced by a decrease in PA intensity, changes in optical extinction, and STEM imaging (Fig. 3, B, C, E, and J), yet in the case of the lower energy pulsed laser test, PA intensity and morphology are well retained (Fig. 3, D and K), further justifying the local surface energy minima hypothesis. The difference in extinction change near 520 nm between GNS and AC-GNS after a high-energy pulsed laser test is very strong evidence of how the AC-GNS platform resists spherical conversion. Further, we see improved PA intensity stability (Fig. 3, B to D) with increasing shell thickness. This proposed stabilization method is also consistent with the thermal stability results (Fig. 3F), which show a substantial blue-shift change in the GNS extinction spectrum compared to the AC-GNS extinction spectrum.

Aside from conferring improved structural stability, the AC-GNS morphology inherently improves PA intensity due to its proportionality to outgoing heat flux into the solvent (*44*). The combined surface area of the hollow shell and internal GNS is greater than that of either shape independently, allowing heat to diffuse more rapidly into the internal and external solvent and subsequently generate a greater PA signal than either shape independently, a phenomenon seen in comparing solid and hollow metallic structures (*45*).

### Whole-body biodistribution of AC-GNS particles via full-view PACT

The AC-GNS morphology and synthesis were designed to optimize passive nanoparticle accumulation within the tumor. First, the particle size falls within the optimal size range (50 to 150 nm) for tumor targeting, notable a balance to avoid rapid clearance or decreased tumor penetration (*46*). Second, the surface of the nanoparticles has been functionalized with SH-PEG_5000_-COOH to increase solution stability and decrease macrophage uptake (*47*), both features that support passive nanoparticle accumulation by extending circulation. The gradual accumulation of nanoparticles from circulation into the tumor causes superior heat contouring around the tumor borders during PTT and has been shown to improve the treatment of irregularly shaped tumors and decrease off-site damage (*48*).

A series of agarose-based phantoms ranging from 7.5 to 120 fmol of AC-GNS particles were imaged using our full-view PACT system (fig. S4, A to C) at 1064 nm and displayed a nearly linear (*R*^2^ = 0.95) increase in the PA signal with increasing particle concentration (fig. S6, A to H). Next, three B6 Albino mice with MB49-derived flank tumors (Fig. 4, A and B) were imaged before and 1, 6, 12, 24, and 36 hours after retroorbital injection of either 300 μg (0.5 pmol), 900 μg (1.5 pmol), or 1500 μg (2.5 pmol) of AC-GNS nanoparticles. Cross-sectional PA images revealed nanoparticle accumulation, signified by increased PA signal intensity throughout the imaging (Fig. 4, C to E, and fig. S7, A to E). The PA intensity of the tumor region of each mouse peaked at the 12-hour time point (Fig. 4F), suggesting that this is when maximum AC-GNS accumulation within the tumor was achieved. The mouse that received the 1.5-pmol dose was then imaged by a separate hybrid PA and ultrasound imaging system using a linear-array ultrasound transducer to validate the results of the full-view PACT (fig. S8, A and B). We also observed an increase in PA signal in lymph nodes following systemic administration, consistent with recent reports that PEGylated nanoparticles of similar size and surface charge can access the lymphatic system via passive drainage or blood-mediated routes (*49*, *50*) and may represent a promising direction for future investigation. As an additional qualitative measure of nanoparticle biodistribution, the same mouse was imaged using a wide-field fluorescence imaging system, in which the fluorescence emission from the dye encased within the AC-GNS particles was recorded (fig. S8, C and D), and was consistent with the PACT results. Hyperspectral SERS scanning (Fig. 4, G to I) was performed on a histological tumor tissue slide to confirm the presence of AC-GNS particles, where the characteristic peak of the HITC dye was detected at 509 cm. These results—supported by phantom-based calibration of PA signal versus concentration, ICP-MS quantification, fluorescence imaging and PA imaging of the same animal using two separate systems, and hyperspectral SERS of tumor histology—confirm that full-view PACT can reliably and noninvasively monitor AC-GNS accumulation and biodistribution.

On the basis of longitudinal PA imaging of the initial cohort, which determined that nanoparticle accumulation within the tumor peaked after 12 hours (Fig. 4F), three additional groups of three mice each were injected with each of the previously mentioned doses. The mean PA intensity within the tumor region was recorded 12 hours after injection (Fig. 4J). While nanoparticle accumulation trends within the tumor roughly followed the injected dose amount, there was substantial variability in the 1.5-pmol group and the average PA intensity of the 1.5-pmol group was quite similar to that of the 2.5-pmol group. This variability is not a limitation of our platform or study design but rather a reflection of the true distribution of biological outcomes encountered in systemic nanoparticle delivery. This point is supported by our ICP-MS analysis of a third cohort of tumor-bearing (*N* = 8), which showed tumor accumulation of 6.7 ± 2.0% initial nanoparticle dose per gram of tissue analyzed (% ID/g) at a single 0.5-pmol dose (fig. S7F), representing nearly 30% relative SD with a cohort size nearly three times larger than those used in Fig. 3J. Factors such as retro-orbital injection efficiency, tumor implantation heterogeneity, tumor-specific vascular architecture, and tumor-size-dependent nanoparticle uptake all contribute to signal variation, even with standardized dosing and imaging (*51*). AC-GNS accumulation was consistent with GNPs of similar size (*52*).

### PA thermometry during PTT

**Fig. 5.**
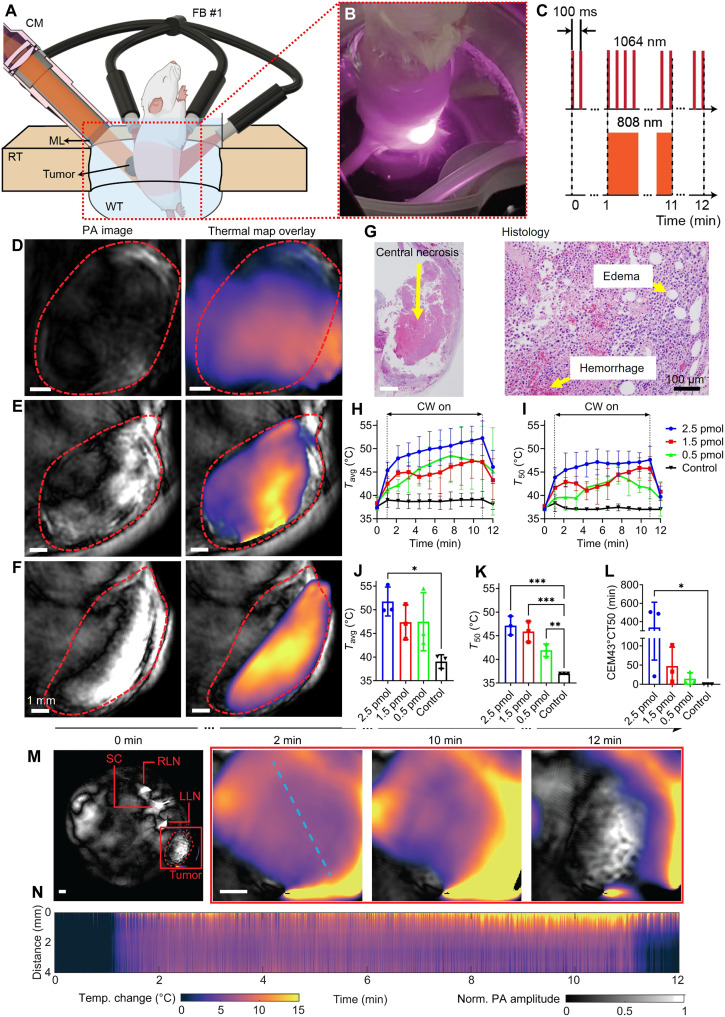
In vivo PA thermometry in PTT. (**A**) Schematic depicting integrated PTT/PACT system. (**B**) Photograph of the experimental setup during PTT. (**C**) Scheme depicting laser emission form PACT and PTT. (**D**) PA image and thermal map recorded during PTT from 0.5-pmol dose group. (**E**) PA image and thermal map recorded during PTT from 1.5 pmol dose group. (**F**) PA image and thermal map recorded during PTT from 2.5-pmol dose group. Red dashed shape denotes tumor region. (**G**) Histology depicting PTT treatment efficacy. (**H**) Average temperature during PTT for each group. (**I**) *T*_50_ for each group during. (**J**) Average temperature of the tumor region after 10 min of PTT. (**K**) *T*_50_ of each tumor region 10 min after PTT. (**L**) PTT CEM43T50 thermal doses for each group. (**M**) Representative PACT image and PA thermal maps with 2.5 pmol at zoomed-in region (red rectangle). (**N**) Spatiotemporal map along the profile line in (M). ML, matching layer; RLN, right lymph node; LLN, left lymph node. Scale bar, 1 mm. *P ≤ 0.05, **P ≤ 0.005, and ***P ≤ 0.0005.

Our full-view PACT system (Fig. 5A and fig. S4, A to C) (*16*) performed cross-sectional thermal mapping during the PTT. We used an AC-GNS–tumor phantom (fig. S9A) to validate the PA thermometry during PTT. A thermocouple was inserted near the center of the spherical AC-GNS–containing region of the phantom, allowing for the simultaneous acquisition of PA thermometry and thermocouple-derived temperature data during single- and multicycle heating experiments (fig. S9, B to E). In both studies, the data recorded via thermocouple had a much steeper slope as compared to the PA thermometry data, which is caused by direct heating of the probe by the continuous-wave (CW) laser (*53*, *54*). To ensure PA imaging and thermometry of the center of the phantom were free of artifacts produced from the thermocouple probe, the probe was slightly offset from the center of the AC-GNS containing region of the phantom, which accounts for the difference in final temperature measured between PA thermometry and the thermocouple seen in the continuous heating experiment (fig. S9C). To assess the photothermal stability of the AC-GNS particles, a multicycle heating experiment was performed (fig. S9D), which shows repeated photothermal responses in both PA thermometry and thermocouple data. To provide a quantitative measure of photothermal stability, we fit the first and last heating cycles of the PA thermometry data and thermocouple data using an exponential rise function

$$T\left(t\right)=a*\left(1-e^{-\mathrm{kt}}\right)+b$$ (1)

where *a* is the heating amplitude or total temperature change, *k* is the thermal rate constant or the speed at which the temperature is changing, and *b* is the baseline temperature. If the particles were deforming, then this would decrease NIR absorption and subsequently cause the amplitude of heating (*a*) and the rate of heating (*k*) to both decrease. The extracted parameters for the PA thermometry data showed excellent agreement between the first and last cycles, where *a*_1_ = 15.865°C, *a*_5_ = 15.806°C, *k*_1_ = 3.104 min^−1^, *k*_5_ = 3.032 min^−1^, *b*_1_ = 0.73°C, *b*_5_ = 10.97°C and R^2^ = 0.99 for both fits. Small percent changes of 0.37% in amplitude and 2.3% in rate constant between the first and last heating cycles further demonstrate the excellent photothermal stability of the AC-GNS platform. The same analysis was performed on the thermal data collected by the thermocouple during the first and last heating cycles, *a*_1_ = 22.826°C, *a*_5_ = 18.229°C, *k*_1_ = 4.422 min^−1^, *k*_5_ = 4.950 min^−1^, *b*_1_ = 0.46°C, *b*_5_ = 9.02°C, and *R*^2^ = 0.99 for both fits. It does not make intuitive sense that there would be a 20.2% decrease in overall heating amplitude, while the heating rate increases by 11.9%. This discrepancy most likely arises from thermocouple-associated heating artifacts. Note that the AC-GNS platform maintains constant heat generation by between the first and last heating cycles during exposure to both pulsed and CW lasers during this study. These data are not only strong evidence of the photothermal stability of AC-GNS particles but also directly address and offer a solution to known thermocouple-induced heating artifacts, namely, PA thermometry, which can provide reliable, noninvasive temperature monitoring with high spatial resolution.

Real-time in vivo thermal mapping (movie S2) of PTT consisting of 10 min of 808-nm laser exposure with a power density of 2.5 W/cm^2^ was performed using the integrated PACT/PTT platform (Fig. 5, A to C). The dose-dependent heat generation during PTT was observed using our integrated platform for 0.5-pmol (Fig. 5D), 1.5-pmol (Fig. 5E), and 2.5-pmol (Fig. 5F) dosages of AC-GNS particles. For each of the three animals, intratherapy thermal mapping was performed and correlated with the histology (Fig. 5G). At all doses, necrosis and edema were observed throughout the tumor region, consistent with the temperature increase measured by PA thermometry during treatment (*55*, *56*). To examine the long-term efficacy of AC-GNS-mediated PTT, three groups of three mice, the same cohort used in Fig. 4G, were each given AC-GNS injections containing either 0.5, 1.5, or 2.5 pmol of nanoparticles 12 hours before PTT. Using intratherapy PA thermal mapping data, we calculated three critical figures of merit used to evaluate the effectiveness of PTT: *T*_avg,_ *T*_50_, and cumulative effective minutes at 43°C 50th percentile (CEM43T50). *T*_avg_ represents the average temperature of the tumor region at a given time, and *T*_50_ represents the temperature of the pixel with the median temperature, a metric that is less sensitive to local hotspots and can provide a more accurate view of tumor heating. CEM43T50 is a thermal dose metric used to normalize treatments that may vary based on the temperature achieved or the duration of therapy and is typically used in clinical hyperthermia trials and treatments (*57*). A mathematical description of the equation can be found in Materials and Methods. Longitudinal *T*_50_ (Fig. 5H) and *T*_avg_ (Fig. 5I) of the tumor cross section exceeded the temperature threshold for cell apoptosis (43°C) for all nanoparticle-receiving groups, whereas none of the animals in the control group (*N* = 3) exceeded the threshold thermal dose value. *T*_avg_ values (Fig. 5J) after 10 min of therapy in all nanoparticle receiving groups were also substantially higher than the control average of 39°C, with group averages of 51.8°, 47.4°, and 47.5°C for the 2.5-, 1.5-, and 0.5-pmol groups, respectively. However, these averages can be severely impacted by the spatial distribution of nanoparticles within the tumor. For example, focal accumulation may yield intense but spatially restricted heating (Fig. 4D), whereas broader distribution (Fig. 4C) supports more uniform thermal profiles. *T*_50_ values (Fig. 5K) taken at the same point are much more consistent within the same group as a result of relying on the median rather than the average temperature. All nanoparticle-receiving groups have a significantly higher median temperature than the control group, with group averages of 47.14°, 45.88°, 41.94°C, and 37.00°C for the 2.5 pmol, 1.5 pmol, 0.5 pmol, and control groups, respectively. The Pearson’s correlation between PA intensity values of the tumor region immediately before PTT (Fig. 4J) with the *T*_50_ value at the 10-min time point (Fig. 5K) to be 0.84 with a *P* value of 0.0007. In addition, a significant increase in CEM43T50 for the 2.5-pmol group was observed compared to the particle-free control group (Fig. 5L).

Using a representative PA thermal map from a mouse in the 2.5-pmol dose group, heat diffusion was tracked throughout the entire tumor region during the PTT period (Fig. 5M). The spatiotemporal temperature map depicted the heat gradient over time provided by continuous PA thermometry monitoring (Fig. 5N). Real-time in vivo thermal mapping using the integrated PACT/PTT platform successfully monitored temperature changes during laser exposure, correlating with histological evidence of tumor necrosis. PACT for real-time PTT thermometry is an effective and precise method for noninvasive thermal dose determination, offering the potential for improved treatment characterization and refinement, ultimately improving treatment efficacy and reducing off-target damage across different cancer types and anatomical locations.

**Fig. 6.**
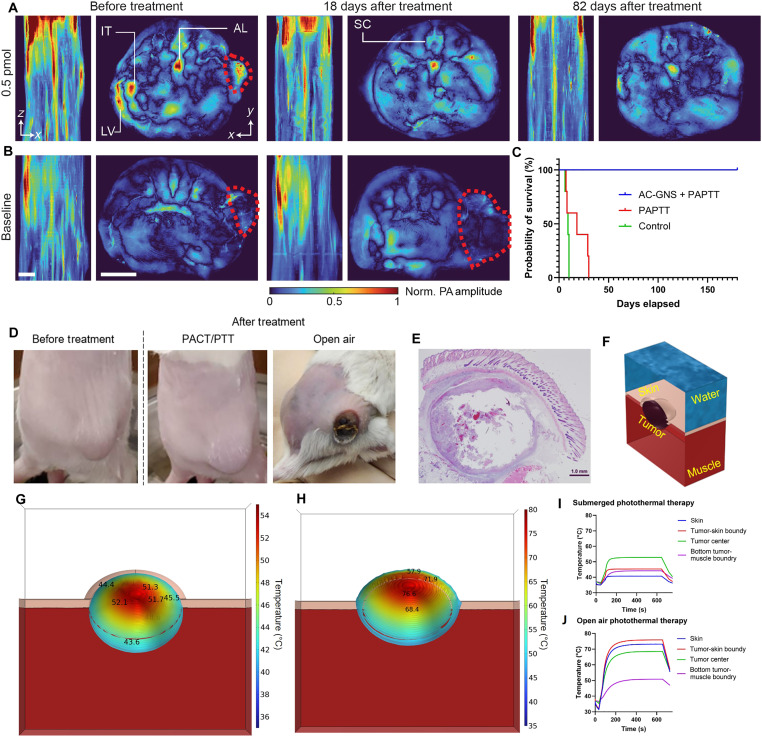
Posttreatment outcomes by the integrated PACT/PTT system. Representative PACT MAPs of the tumor region before and after treatment at different time points of (**A**) 0.5 pmol and (**B**) the control group. (**C**) Survival curve of the AC-GNS–mediated PACT/PTT (AC-GNS + PAPTT) (2.5 pmol, *N* = 3. 1.5 pmol, *N* = 3. 0.5 pmol, *N* = 3), PTT without AC-GNS (PAPTT) (*N* = 5), and control group (*N* = 5). (**D**) Photographs of the tumor before and after treatment with the PACT/PTT system and with the open-air setup. (**E**) H&E histology of a representative tumor slice in the control group with integrated PACT/PTT. (**F**) Cross-sectional view of the simulation model of the tumor temperature after 10 min of PTT with (**G**) the PACT/PTT setup under water and (**H**) the open-air setup. The simulated temperature measurements over 10 min of PTT with (**I**) the PACT/PTT setup and (**J**) the open-air setup. IT, intestine; AL, aortic lumbar lymph node; LV, lymphatic vessel.

PACT images with the tumor region outlined in red reveal complete tumor regression only 18 days following treatment for a representative member from the 0.5-pmol dose group (Fig. 6A). In contrast, the tumor of a nanoparticle-free mouse more than doubled in size over the same period, requiring the sacrifice of the animal (Fig. 6B). All mice (*N* = 9) that received AC-GNS particles at any dose survived symptom-free for 6 months following treatment, resulting in a 100% survival rate (Fig. 6C). In contrast, all animals (*N* = 5) that did not receive treatment reached humane end points by day 10, and all animals (*N* = 5) that only received laser exposure reached humane end points by day 30. All long-term survivors were euthanized at the end of the 6-month survival study, at which point gross necropsies were unremarkable. Key hepatotoxicity markers were not significantly different from those in animals that did not receive the nanoparticle injection (fig. S10, A to C) and were within the expected ranges of coisogenic strains, as reported by Charles River Laboratories. This good long-term biocompatibility of AC-GNS particles is consistent with initial safety evaluations of gold nanoshells with similar size and surface modification in humans (*58*). On the basis of elimination studies of gold nanoparticles of a similar size, AC-GNS particles are most likely eliminated via the hepatobiliary system over the course of weeks and months (*59*). Furthermore, all long-term survivors rapidly recovered after photothermal therapy, increasing their body weight until the end of the study (fig. S10D).

In addition, we suspect that by submerging the tumor region underwater to enable real-time intratherapy PA thermometry, the water bath acted as a heat sink, protecting the skin and surrounding nontumor structures from unintended thermal damage. Significant edema induced by the AC-GNS–mediated PTT was noted following treatment however, for the 0.5- and 1.5-pmol dose groups, little to no skin damage was observed (Fig. 6D), confirmed by histology (Fig. 6E). Further, all animals that received AC-GNS–mediated PTT ambulated normally immediately after waking up from anesthesia after therapy. In contrast, significant skin damage was observed under identical conditions when open-air PTT was performed (Fig. 6D).

To further test this hypothesis, we developed a two-step simulation scheme where identical models consisting of the skin, muscle, nanoparticle-containing tumor region, and either air or water were constructed in MCmatlab (*60*) (Monte Carlo) and COMSOL Multiphysics to model AC-GNS–mediated PTT of a mouse flank tumor (Fig. 6F). Monte Carlo simulations were used to calculate the optical fluence (fig. S11A) and absorbed power (fig. S11B) throughout the simulated PTT model based on the literature derived optical properties of tissue and water. The resulting power deposition matrix was then imported into COMSOL and used as a heat source. The bioheat transfer module was used to assign tissue-specific physical properties based on the material library and literature-derived tissue-specific perfusion rates. All boundary conditions were set to reflect experimental conditions, which are described in detail along with model dimensions and the tissue-specific properties in Materials and Methods.

Thermal contour maps after 10 min of simulated treatment revealed that the location of the maximum temperature achieved during PTT occurred much closer to the center of the tumor in the case of submerged PTT (Fig. 6G) compared to open-air PTT, where the location of the maximum temperature achieved was much closer to the skin surface (Fig. 6H). The point temperature readings (Fig. 6, I and J) show the change in temperature over time in the center of the skin layer, tumor-skin boundary, center of the tumor, and bottom tumor-muscle boundary for both submerged and open-air PTT, respectively. For the open-air PTT case, the highest temperature recorded occurred at the tumor-skin boundary, closely followed by the skin temperature, center of the tumor, and tumor-muscle boundary. In contrast, the highest recorded temperature for the submerged PTT model occurred at the center of the tumor, followed by the tumor-skin boundary, tumor-muscle boundary, and last, the skin. The near-complete inversion of the heating trends observed between the two treatment types was due to the surface cooling provided by the water in the case of submerged PTT, illustrating the possible skin-saving utility of this integrated treatment platform. There are several reasons for the relative improvement seen in long-term survival presented in this work compared to similar investigations using systemically delivered GNS for PTT performed by our group and others in the past (*61*, *62*). First, the improved photostability and subsequent reliable photothermal performance of the AC-GNS platform allowed us to use lower systemic doses to achieve the desired therapeutic out. Second, the specific design choices of particle size and surface functionalization were selected to maximize passive uptake into the tumor region. Last, off-target tissue damage was significantly reduced by performing treatment underwater, which decreased the overall burden on the mice and enabled them to make a full and rapid recovery.

## DISCUSSION

This work addresses key obstacles currently hindering nanoparticle-mediated PTT treatment design and investigation by (i) developing a stable, novel nanoparticle platform that is a sensitive contrast agent and achieves a robust therapeutic effect and (ii) integrating PTT with real-time noninvasive thermal monitoring of tumor regions during therapy. The tunable AC-GNS particle morphology delivers exceptional thermal stability under physiological conditions (37°C) and photostability under pulsed laser exposure required for PACT, outperforming conventional GNS particles while retaining solution stability and excellent NIR absorption. This improved stability prevents the blue shifting of the nanoparticle extinction spectrum, ensuring that the contrast agent and photothermal transduction performance of the AC-GNS remain constant from the time of injection to the time of PTT, even after multiple PACT measurements to monitor particle biodistribution and intratherapy thermal monitoring via PACT.

By leveraging the increased stability of the AC-GNS nanoparticle platform, we performed noninvasive, real-time biodistribution monitoring of particles down to the femtomole regime using a full-view ring-array transducer PACT system. This effective integration allowed us to perform deep tissue and noninvasive thermal monitoring of the tumor region during PTT. This methodology provides a broadly applicable approach for precisely guiding and evaluating thermal therapies, generating deep-tissue thermal maps of tumor regions, and allowing accurate prognostic thermal dose determination. Histological analysis was consistent with the PA thermometry results, revealing that heat-induced tumor cell death was directly proportional to the thermal dose. There was a 100% success rate in generating long-term survivors with no signs of injury or toxicity for all AC-GNS dosages tested. Thermal dose and long-term survival data support the notion that thermal data recorded during PTT via PACT contains prognostic information for long-term treatment outcomes, underscoring the potential of real-time PACT thermometry enabled by stable nanoparticles to guide treatment and predict therapeutic success, which is valuable in preclinical cancer therapy research.

This work also demonstrates that effective PTT without substantial skin damage can be achieved by leveraging the aqueous coupling medium necessary for PACT as a natural heat sink that facilitates skin surface cooling and protects the surrounding nontarget tissues from heating. The methods outlined in this work can be used for noninvasive and repeated evaluations of the effects of nanoparticle design on accumulation within the TME and the in vivo performance of other PTT treatment schemes. The synergistic integration of our AC-GNS–mediated PTT/PACT system establishes a strong foundation for advancing cancer therapy research and the future clinical translation of innovative imaging-guided treatment and monitoring strategies.

The current preclinical system is ideally poised to advance oncology research in small-animal models by leveraging the advantages of AC-GNS performance, full-view PACT imaging, and thermometry. These investigations could explore different cancer models, active nanoparticle targeting strategies that have been shown to improve nanoparticle accumulation by up to 50% (*52*), adaptive optic-integrated CW laser delivery to further optimize thermal dose, thermal dose-dependent immune responses to treatment, and combinations of photothermal immunotherapy. In the future, we envision a clinical PTT/PACT system using a linear-array or matrix-array transducer, which will further reduce costs and improve positioning for treatment (*63*). Such a module should be effectively combined with a miniaturized water circulation bath for acoustic coupling and thermal dissipation effects. In addition, the future implementation of a thermal memory-based method, which is independent of calibration with known tissue properties, would enable absolute temperature measurement via PA thermal conversion (*16*). These advancements, combined with automated nanostar synthesis systems (*64*), will enable the investigation of this theranostic platform in larger animal models and ultimately aid in clinical translation.

This study presents solutions to the current obstacles hindering the investigation and refinement of preclinical light-based cancer therapies by integrating AC-GNS–mediated PTT with full-view PACT-enabled real-time nanoparticle tracking and precise thermal monitoring in deep tissues using a murine bladder cancer model, resulting in a 100% survival rate. The integration of these technologies has resulted in a theranostic platform that elevates the standards for imaging-guided nanoparticle-mediated PTT and provides the tools necessary to investigate personalized adaptive cancer therapies that combine precision treatment with real-time monitoring for superior outcomes.

## MATERIALS AND METHODS

### AC-GNS synthesis, characterization, and modeling

The precursor bimetallic nanostar particles were synthesized according to the method described by Fales *et al.* (*65*). To synthesize ~100 ml of bimetallic nanostar particles, 1 ml of a 12-nm gold sphere solution with an optical density of 2.86 at 520 nm was added to 100 ml of 25 μM gold chloride. Then, in rapid succession, 500 μl of 2 mM silver nitrate, 500 μl of 0.1 M ascorbic acid, 500 μl of 0.1 M silver nitrate, and 100 μl of ammonium hydroxide was added. After 1 hour, 10 ml of PVP solution (55 mg/ml) was added to the bimetallic nanostar solution and mixed for 30 min. Particles were centrifuged, concentrated in deionized (DI) water, and stored at 4°C until further use.

To prepare ~100 ml of AC-GNS particles, the previously prepared bimetallic nanostar particles were concentrated to a final volume of 5 ml and resuspended in 60 ml of 0.1 M ascorbic solution. A PVP solution [30 ml, PVP (10 mg/ml)] was added to the mixture, followed by 500 μl of sodium hydroxide. Using a syringe pump, 2 ml of a 5 mM gold chloride solution was added for 30 min. The gold-coated bimetallic nanostar particles were centrifuged and resuspended in 5 ml of PVP solution (10 mg/ml). To initiate the galvanic replacement-free removal of the silver, the concentrated particle solution was added to a 60 ml of PVP solution (10 mg/ml) containing 500 μl of hydrochloric acid and 10 ml of 30% hydrogen peroxide solution at 55°C. After 45 min, the unsealed C-GNS particles were centrifuged with DI water and ethanol to remove insoluble silver species.

To facilitate dye loading into the hollow region of the particles concentrated in ethanol, 1 mg of HITC dye was added and mixed with the particles for 1 hour. The concentrated nanoparticle solution was added to 80 ml of PVP solution (10 mg/ml) containing 100 μl of hydrochloric acid and 500 μl of 0.1 M ascorbic acid. Gold chloride solution (5 mM) was then added at a rate of 1 ml/10 min, for a volume of 1 ml in the case of AC-GNS-1 particles to 5 ml in the case of AC-GNS-5 particles.

After AC-GNS formation, particles were washed with DI water and ethanol to remove any surface-bound PVP or dye molecules and then were incubated in 10 ml of ethanol containing 10 mg of SH-PEG_5000_-COOH at 45°C overnight. Particles were centrifuged, resuspended in PBS, and stored at 4°C until further use. Nanoparticle extinction spectra were recorded using a Shimadzu UV-3600i system. SERS spectra were recorded using a WP 785X-ILC system (Wasatch Photonics). High-angle annular dark-field–STEM and STEM-EDS were performed using a Talos F200X instrument. Hyperspectral SERS scanning of the histological slides was performed at 785 nm using a Horiba Jobin Yvon LabRam ARAMIS system. Nanoparticle tracking analysis was performed using the NanoSight500 system (Malvern Panalytical). To prepare AC-GNS phantoms, a 3% OmniPur agarose solution was brought to a boil and was used to fill three-dimensional (3D) printed phantom molds. Small aliquots of agarose solution were then allowed to briefly cool to ~60°C and mixed with varying concentrations of AC-GNS solution. The ungelled AC-GNS agarose solution was then pipetted into the vacant center of the agarose phantom and stored in DI at 4°C until use.

All optical nanoparticle simulations were performed using COMSOL Multiphysics 6.0 and the wave optics package. The constant underlying GNS morphology had a core radius of 15 nm, a branch length of 25 nm, a lower branch radius of 4 nm, and a branch tip radius of 2 nm. The internal water layer with the AC-GNS model had a thickness of 20 nm. The material properties of gold were selected on the basis of the values provided by McPeak *et al.* (*66*) and the surrounding water by Querry *et al.* (*67*). All domains of the simulations were meshed using extremely fine settings.

### EELS analysis

AC-GNS particles were dispersed on ultrathin carbon support films on copper support grids and cleaned for 30 s in Argon plasma. EELS was performed in STEM mode using a Thermo Fisher Titan 80-300 operated at 80 kV and equipped with a Gatan Enfinium ER EELS spectrometer. A monochromated electron beam with an energy resolution of 0.15 eV was used for imaging and EELS. Data were acquired with a spectral dispersion of 0.01 eV per channel. During EELS acquisition, a convergence semi-angle of 19.3 mrad, collection semi-angle of 71 mrad, and an entrance aperture of 5 mm were all used. A spectrum image of a portion of an AC-GNS particle was acquired using these settings, with a size of 74 × 39 pixels. Following best practices for EELS data analysis (*38*), the zero-loss peak of all pixels was first aligned to zero. Then, all spectra were normalized so that the zero-loss peak intensity for all spectra was the same. Using the built-in statistical tools in DigitalMicrograph software, a PCA decomposition analysis was performed on the spectrum image data, and the top eight components were selected for reconstruction. Next, while still in DigitalMicrograph software, a varimax rotation was performed in the spatial domain using the top eight components, which, after inspection, were all selected for reconstruction. After data denoising, the tail of the zero-loss peak was fit with a logarithmic function between 0.45 and 0.95 eV and subtracted as background for the entire spectrum image (*38*). Locations of EELS spectra 1, 2, and 3 were selected on the basis of the distribution of representative spatially trackable spectral components. Background-subtracted spectra were exported from DigitalMicrograph, and then Lorentzian curve fitting and statistical analysis were performed using Python. The entire background-subtracted spectrum image was exported to Python to generate movie S1. A nonlinear median filter was applied to images of the non-negative scores of representative spatially trackable spectral components and the LDOS at 1.58 eV to improve image quality.

### Monte Carlo simulations of PACT imaging

The MCXLAB simulation package, the native MEX version of MCX for MATLAB, was used to perform a Monte Carlo simulation to model the photon propagation during laser excitation from the ring-array PACT system (*68*). The Digimouse atlas was used to define optical properties for a mouse model in the simulation for varying tissues and organs (*69*). The labels defined in the model were water, bone, adipose tissue, heart, muscle, stomach wall, liver, spleen, kidneys, lungs, and skin. The label model had an isotropic voxel size of 0.3 mm. Four optical properties were defined for each label in the model: absorption coefficient, scattering coefficient, scattering anisotropy, and refractive index. The scattering and absorption coefficients for each tissue type were calculated using the method described by Alexandrakis *et al.* (*70*) for λ = 1000 nm. The scattering anisotropy and refractive index were defined as 0.9 and 1.3 for each label in the model, respectively. Eight simulations were performed independently and subsequently to model the light output from each of the eight fibers. For each simulation, a cone beam with a half-angle of π/6 , an inclination of 30°, and a distance of ~1 cm from the mouse skin surface was defined as the light source (modeling the true ring-array system light source). One hundred million photon packets were modeled for each simulation, with a temporal resolution of 1 ps for a duration of 120 ps. Following the eight simulations, the resulting 3D fluence distribution was summed to generate a final fluence distribution of the combined eight-fiber excitation.

### Full-view PACT system construction

The full-view PACT system consisted of three main components: a pulsed laser, ultrasonic transducer, and data acquisition (DAQ) unit. For the PA signal excitation, we used a 1064-nm pulsed laser (Quantel DRL) with a 10-Hz repetition rate and 10-ns pulse width. Laser pulses were delivered through two fiber bundles (Dolan-Jenner Industries), each with four outlets (Fig. 4A and fig. S4A). The output from the eight fiber outlets formed a uniform surrounding illumination around the mouse (Fig. 4B and fig. S4A). Upon PA signal generation, full-view detection of acoustic waves is conducted by a ring-array transducer (IMASONIC SAS) comprising two half-ring probe (5-MHz center frequency, 40-mm radius, 0.47-mm pitch, 0.1-mm element spacing, and 512 elements in total). Each element had an elevational focus (0.17 numerical aperture) with 45-mm focal distance and ~10-mm depth of focus. Thus, a pair of opposite elements forms a one-way −6-dB focal zone of 20-mm diameter at the center of the transducer. The received acoustic signal from the transducer was preamplified using four amplifiers (LEGION AMP, PhotoSound Technologies Inc.) before being digitized using a 256-channel DAQ system (Vantage 256, Verasonics Inc.). The PACT system had 150-μm in-plane resolution of ~1.5-mm elevational resolution of (fig. S12, A and B). Thus, the PA signals from the 512 elements were multiplexed using two laser pulses for one PA image. Laser firing and data acquisition were synchronized using an embedded device with a field-programmable gate array (FPGA) (myRIO 1900, National Instruments).

For animal positioning in the integrated PACT/PTT system, the hind legs of the mouse were taped into a bottom holder, and the front legs were taped into the anesthesia mask. The bottom holder and mask were connected to a motorized translation stage. The entire body of the mouse, excluding the head, was submerged in a water chamber to provide acoustic coupling for the PACT. The water was circulated through a tube in a heating bath to maintain water temperature of 37°C.

For whole-body 3D imaging, the animal was vertically passed through the center of the ring-array using the translational stage (Fig. 4, A and B and fig. S4B). All 3D scans covered 5-cm scanning range, with 0.2-mm step size and 1000 frames in total. At each position, four PA frames were acquired. To mitigate motion artifact between each step, we measured cross-correlation between the four frames of the next step with the chosen frame of the current step. The chosen frame of the next step was the one with highest cross-correlation peak value. During 2D PA thermometry combining with PTT, the animal was in a fixed position.

### Animal model preparation

All animals used in this study were B6 albino mice between 6 and 8 weeks of age sourced from Charles River Laboratories. All animal studies were performed in accordance with the protocol approved by the Institutional Animal Care and Use Committee of the Duke University. Duke University’s Animal Care and Use program is fully accredited by Association for Assessment and Accreditation of Laboratory Animal Care International, registered as a research facility with the US Department of Agriculture in accordance with the Animal Welfare Act and all amendments and holds Category 1 Assurance with the Public Health Service (through the NIH’s Office of Laboratory Animal Welfare). To prepare tumors, 2.5 × 10^6^ MB49 (ACCEGEN, ABC-TC223S) cells suspended in 50% Matrigel were injected into the flank. Nanoparticles were administered via retroorbital injection when the tumor volumes were 100 and 200 mm^2^. All retro-orbital injections were performed in a volume of 50 μl. Animals with tumor volumes within the desired range were randomly divided into treatment groups. The humane end points of failure to thrive or tumor volume exceeding 1000 mm^2^ were used in this study. Animals were anesthetized using vaporized isoflurane and euthanized using CO_2_.

### Integration of PTT with PACT

The key step in integrating PTT with the full-view PACT system is the light delivery of the CW laser in the PACT setup (Fig. 5, A and B). A CW laser (Opto Engine LLC) was coupled through a fiber connected to a collimator (F810SMA, Thorlabs Inc.). The collimated light then follows a tunnel whose central axis intersects the perimeter of the transducer’s −6-dB focal zone, where the tumor is positioned. The light tunnel has a diameter of 15-mm. It was larger than the beam size (10 mm), allowing for further adjustment of the laser targeting the tumor. The end of the tunnel toward the transducer was enclosed by a thin agar layer (~3-mm thickness). It serves as a matching layer to provide a normal incidence angle at the air-water interface. Before each treatment, real-time PA imaging allows us to precisely position the mouse and tumor with the CW laser, as we know where it intersects the imaging plane owing to the light tunnel.

The total duration of each treatment session with PA imaging was 12 min (Fig. 5C). The first and last minutes served as the baseline when the CW laser was switched off. In the middle (10 min), the CW laser was switched on for PTT. During the entire 12-min period, PA images were continuously acquired using a 1064-nm pulsed laser. PTT illumination and PACT acquisition were synchronized by the embedded device with the FPGA (myRIO 1900, National Instruments). A gel tumor phantom was heated using the same time sequence to validate PA thermometry using a thermometer. Following the continuous heating study used to approximate the treatment, the laser was then cycled between the on and off states every 30 s to test the repeatability of the PA thermometry and the photostability of the AC-GNS particles.

### Real-time matrix-multiplication PACT image reconstruction

The core of PACT image reconstruction is the half-time delay-and-sum (DAS) (*71*–*73*). Briefly, DAS relies on the calculation of the arrival time of acoustic waves. The time delay is then indexed in the RF data to backproject the signal at the corresponding time. This process was repeated for all data channels until the final PACT image was formed. Half-time DAS further reduces the artifact of the traditional method by truncating half of the RF data, which mostly contains the reflected signal (*71*). The reconstruction is implemented using sparse matrix multiplication (*74*), allowing real-time PA imaging at a laser repetition rate of 10 Hz.

### PA signal processing pipeline

Raw RF data of the received signal and the reconstructed image was processed in two ways, depending on the end point of quantification (fig. S12C). To quantify the biodistribution of whole-body scanning data, no frequency filter was applied to the raw data. Upon reconstruction, we simply took the absolute values of the reconstructed bipolar slices in the entire 3D volume. To generate the PA thermal map, the raw data were processed following low-frequency signal extraction, which has been shown to improve PA thermal tracking (fig. S12C) (*75*). First, RF data from each channel are deconvolved with the electrical impulse response to approximately recover the receiving bandwidth, particularly low-frequency signals (*75*). Then, a low-pass filter with an empirical 0.5-MHz cutoff frequency was applied to the deconvolved data to extract low-frequency components for reconstruction (*75*). We further implemented image registration to PA frames during PTT because the PA thermal map of the relative temperature change depends on the stable position of the tumor over time (fig. S12C). The geometric transformations for image registration include rotation and translation. The registered PA frames were then ready for subsequent thermal conversion for PA thermometry.

### Deep tissue PA thermometry and thermal dose calculation

PA thermometry is a spatial map of the relative temperature change converted from the relative signal change between PA frames (*14*). This thermal conversion is enabled by the relationship between the initial PA pressure increase $p_{0}$ and the Grüneisen parameter $\Gamma_{0}$

$$p_{0}=\Gamma_{0}\eta_{\text{th}}A_{e}$$ (2)

where $A_{e}$ is the specific optical absorption and $\eta_{\text{th}}$ is the thermal conversion percentage (*76*). The relationship between $\Gamma_{0}$ and the baseline temperature $T_{0}$ is (*15*)

$$\Gamma_{0}=bT_{0}+c$$ (3)

where b and c are empirical constants based on linear fitting between $\Gamma_{0}$ and $T_{0}$ of the associated sample. From Eqs. 2 and 3, we can derive the relative PA signal change with respect to the temperature change ∆T as (*15*)

$$\frac{∆p}{p_{0}}=\frac{b∆T}{bT_{0}+c}$$ (4)

Thus, PA thermal map of relative temperature change can be expressed as (*77*)

$$∆T=a\frac{∆p}{p_{0}}$$ (5)

$$a=T_{0}+\frac{c}{b}$$ (6)

For phantom, $T_{0}=22{}^{\circ}C$ based on water temperature and $\frac{c}{b}=2.75$ based on experimental calibration from Bakaric *et al.* (*78*, *79*). For soft tissue, $T_{0}=37{}^{\circ}C$ based on body temperature and $\frac{c}{b}=13.14$ from Zhou *et al.* (*16*). Subsequently, in vivo PA thermal map was derived simply by adding body core temperature to the relative temperature change (∆T+37°C).

For thermal dose calculation, we used the cumulative equivalent minutes at 43°C at *T*_50_ (CEM43T50), a widely used metric in hyperthermia clinical trials (*57*). The formula for CEM43T50 is (*57*)

CEM43T50=∑i=0ntR43−T50i{R=0.5 for T50i>43°CR=0.25 for T50i>43°C (7)

Here, *t* is the PA imaging period, *n* is the total duration of PTT when the CW laser is on (10 min), and T50*_i_* is the temperature that exceeds 50% of the tumor region-of-interest (ROI) in the *i*th thermal map (median of all pixels in the thermal map) during the 1-min time interval (*57*). In addition to CEM43T50, the average temperature of the tumor ROI (*T*_avg_) was considered for thermal quantification.

### Monte Carlo and PTT simulations in COMSOL

Monte Carlo simulations were performed using an open-source MCmatlab solver (*60*). The dimensions of the simulated region were 2 cm in all directions. The simulated beam profile was designed to match the beam profile used experimentally, in which a Gaussian beam with a radius of 16 mm was passed through an iris of 9.6 mm, which resulted in a simulated power density of 2.5 W/cm^2^. The muscle layer had a height of 1.1 cm, and the skin had a thickness of 0.075 cm. The tumor was designed on the basis of experimental observations and was modeled as an ellipsoid with radii of 3.5, 4, and 2.5 mm, resulting in a tumor volume of 141 mm^3^. Absorption and scattering coefficients of 0.1 and 100 cm^−1^ were assigned to all tissue regions to approximate the optical properties of generic tissues (*76*). An absorption coefficient value of 0.02 cm^−1^ was assigned to the water layer in the case of submerged PTT (*67*). The absorption coefficient of the tumor region was increased by 1.36 cm^−1^ based on the average % ID/gram as determined via ICP-MS for the median dose of AC-GNS particles used in this study and assuming the even distribution of particles throughout the tumor region. Monte Carlo simulations for both open-air PTT and submerged PTT were performed identically to experimental PTT.

A model identical to the simulated Monte Carlo model was constructed in COMSOL Multiphysics 6.0 to simulate the heat generation caused by simulated photothermal therapy. A bioheat transfer physics package was applied to the entire model. Tissue-specific thermal properties were assigned based on the material library of the bioheat transfer physics package. All tissues had an initial temperature of 37°C, ambient air temperature was set to 20°C in the case of open-air PTT, and the surrounding water temperature was set to 35°C in the case of submerged PTT. COMSOL material definitions were used to assign the thermal properties to all phantom domains. The boundary conditions of 37°C were applied to all tissue-adjacent surfaces, 20°C for all air-adjacent surfaces, and 35°C for all water-adjacent surfaces. Tissue-specific perfusion rates based on the work done by Hall *et al.* were applied to all applicable domains (*80*). The perfusion rate of the muscle was also applied to the simulated tumor region. The power deposition matrix derived from the Monte Carlo simulations was exported to COMSOL and used as the heat source for the PTT duration.
