# Supplementary material for: Advancing precision photothermal therapy by integrating armored gold nanostars with real-time photoacoustic thermometry and imaging
Source: Sci Adv. 2025 Aug 13;11(33):eadx6350. doi: 10.1126/sciadv.adx6350 (PMC12346267; doi:10.1126/sciadv.adx6350)
Supplement: Supplementary file 1 — Figs. S1 to S12 Legends for movies S1 and S2 [file sciadv.adx6350_sm.pdf]

Science Advances
AAAS

Supplementary Materials for

Advancing precision photothermal therapy by integrating armored gold nanostars with real-time photoacoustic thermometry and imaging

Aidan J. Canning *et al.*

Corresponding author: Junjie Yao, junjie.yao@duke.edu; Tuan Vo-Dinh, tuan.vodinh@duke.edu

Sci. Adv. **11**, eadx6350 (2025)
DOI: 10.1126/sciadv.adx6350

The PDF file includes:

Figs. S1 to S12
Legends for movies S1 and S2

Other Supplementary Material for this manuscript includes the following:

Movies S1 and S2

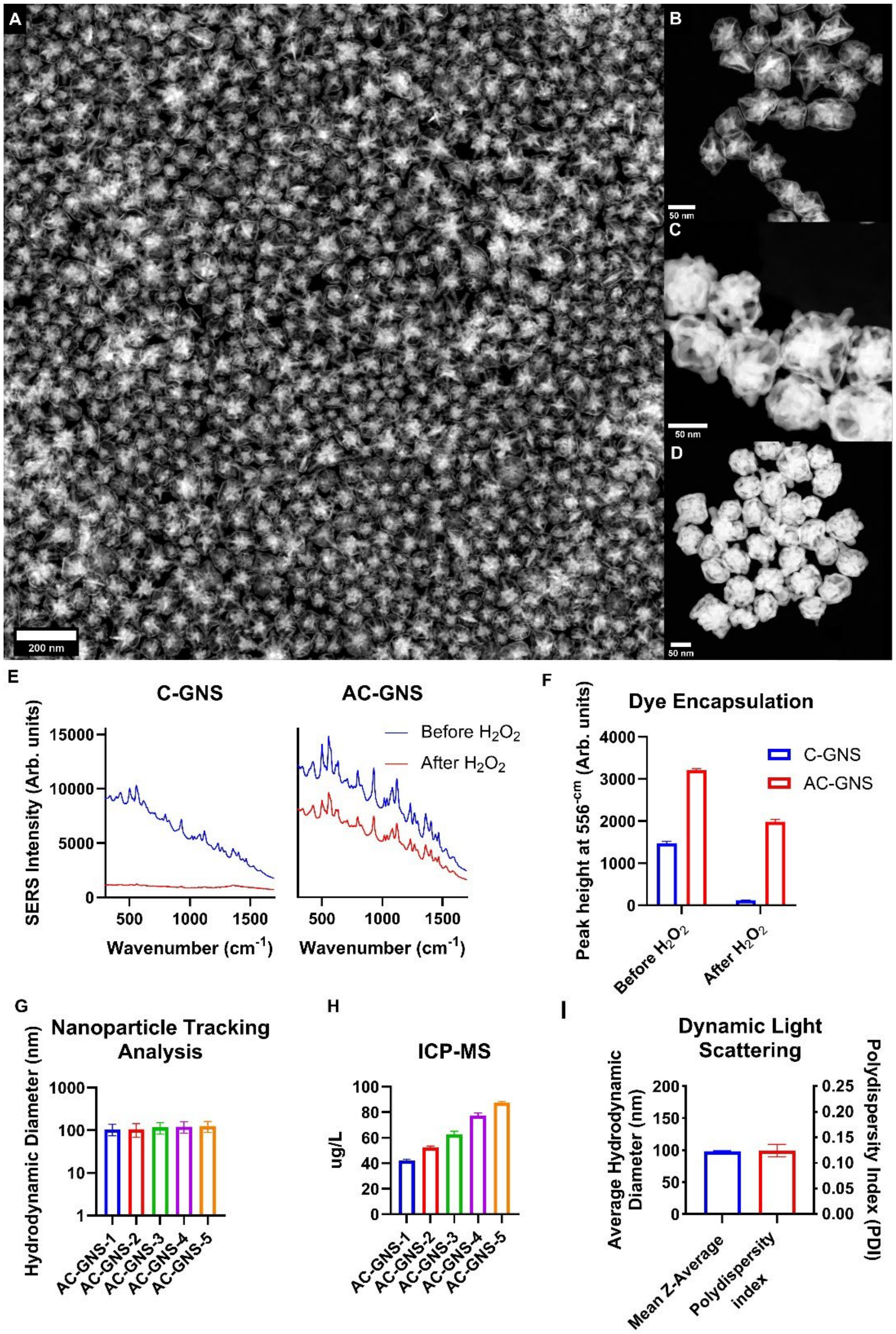

Fig. S1- Additional AC-GNS characterization. (A) Unsealed caged gold nanostars. (B) AC-GNS-1 particles. (C) AC-GNS-3 particles. (D) AC-GNS-5 particles. (E) SERS signal of C-GNS and AC-GNS particle before and after 30 minutes in 6% H_2O_2 solution, N=5. (F) Quantification of change in SERS peak height for C-GNS and AC-GNS particles before and after 30 minutes in 6% H_2O_2 solution, N=5. G) Nanoparticle tracking analysis results for different AC-GNS formulations, N=3. H) ICP-MS analysis of gold concentration for each AC-GNS morphology, N=3. I) Mean hydrodynamic diameter and polydisperity index of AC-GNS particles determined via DLS (N=5).

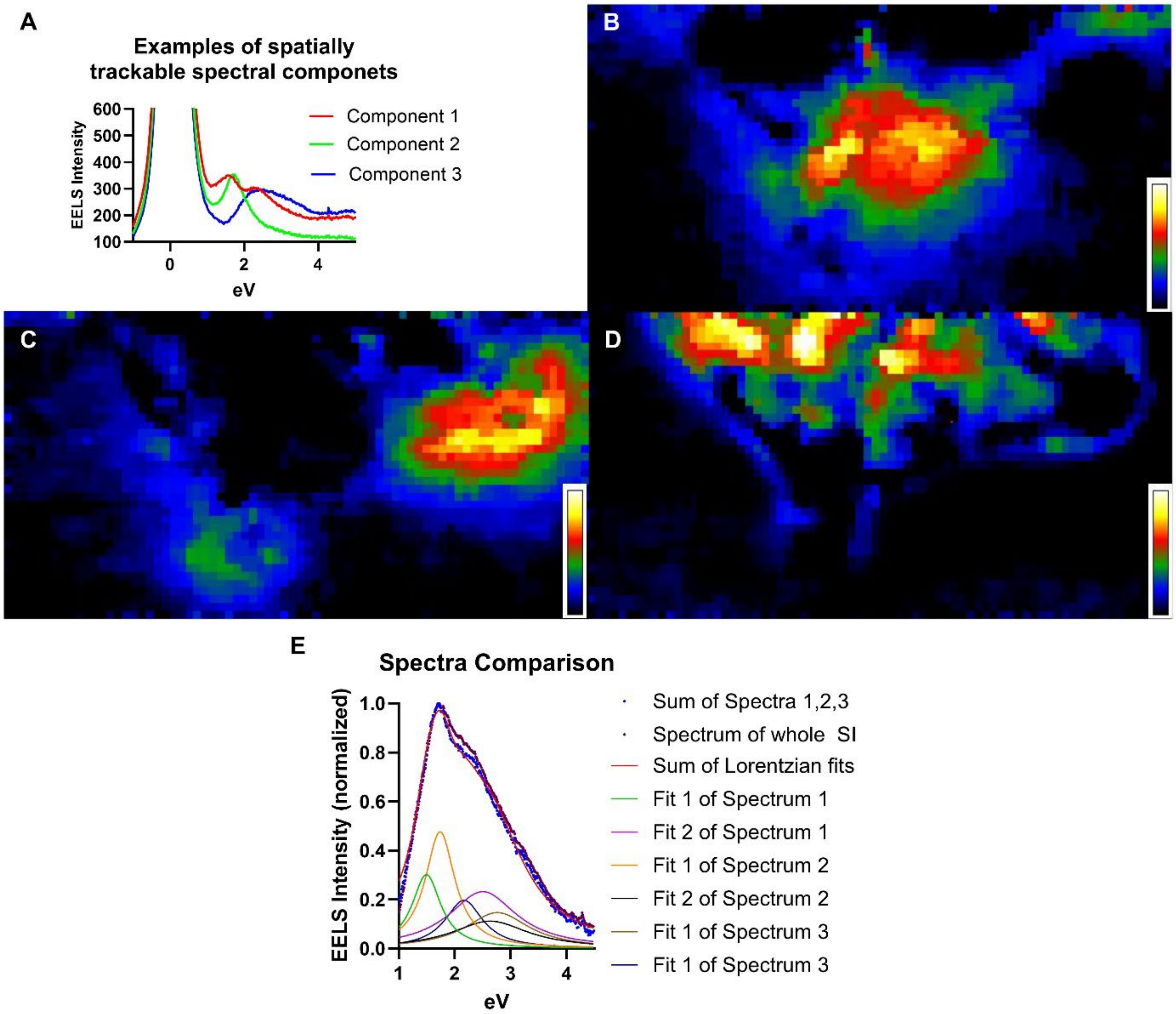

Fig S2. EELS analysis of AC-GNS particle. (A) Examples of spatially trackable spectral components generated from a varimax rotation performed in the spatial domain. (B) Non-negative score of component 1. (C) Non-negative score of component 2. (D) Non-negative score of component 3. (E) Comparison of all EELS spectra and Lorentzian fits.

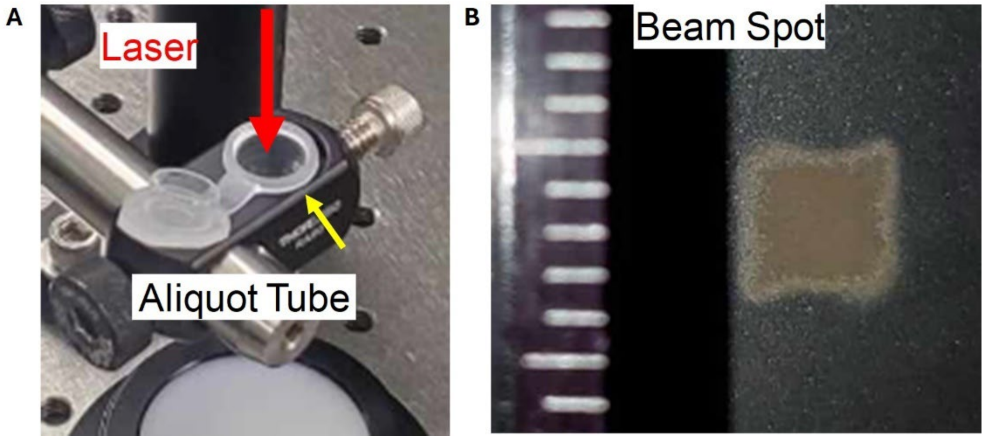

Fig S3. Photostability test for AC-GNS and GNS at 110 mJ/cm^2. (A) Test setup with free-space laser illumination into an aliquot tube holding the nanoparticle samples. (B) Beam spot of the incident laser, showing an area of ~0.09 cm^2.

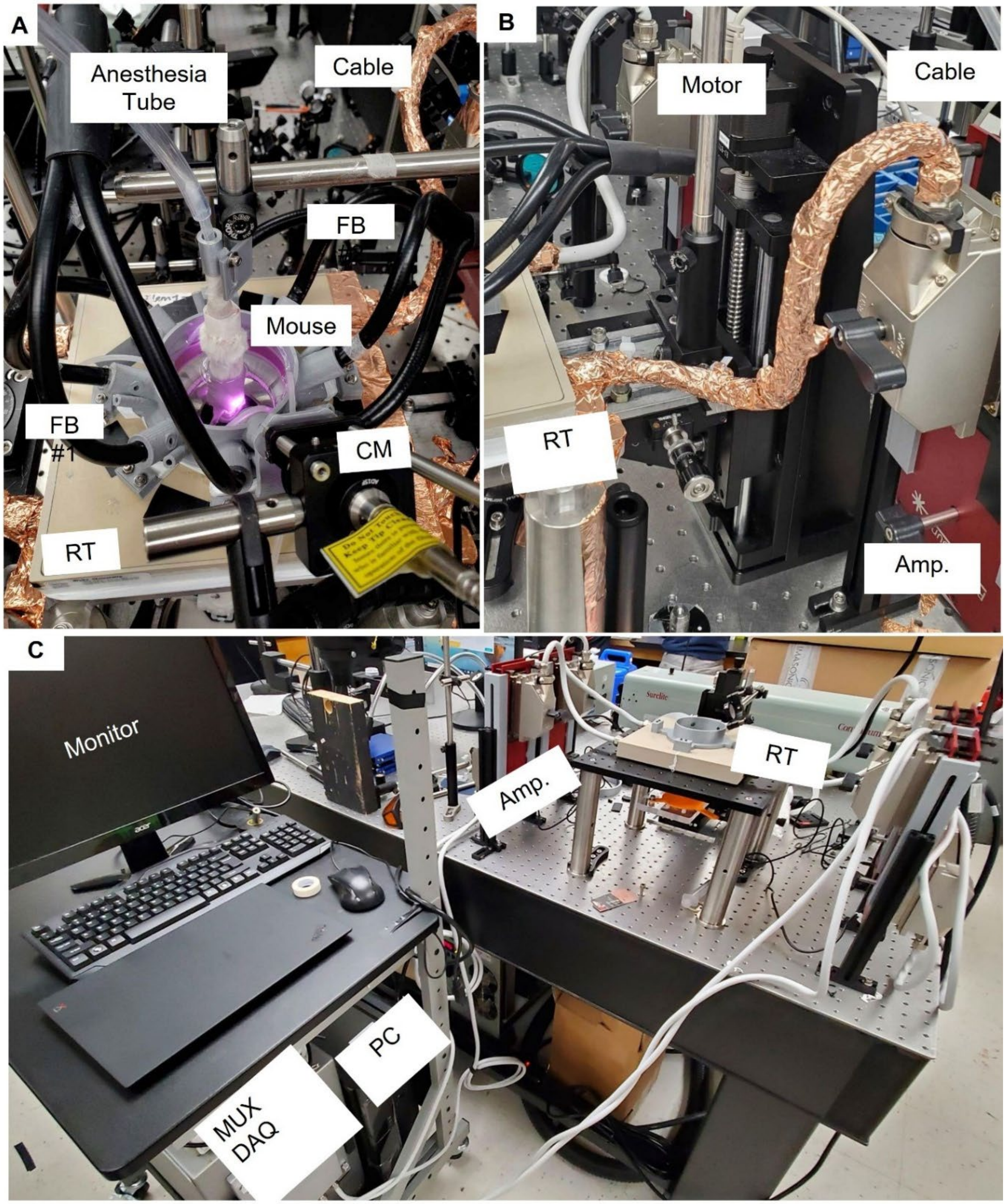

Fig S4. Photographs of the full-view PACT system. (A) *In vivo* experimental setup showing the CW laser heating during PTT. During whole-body 3D scan, CW laser is not turned on. (B) Translational stage for mouse scanning. (C) Wide view of the system. FB, fiber bundle; RT, ring-array transducer; CM, collimator; DAQ, data-acquisition instrument; MUX, multiplexer; Amp., amplifier.

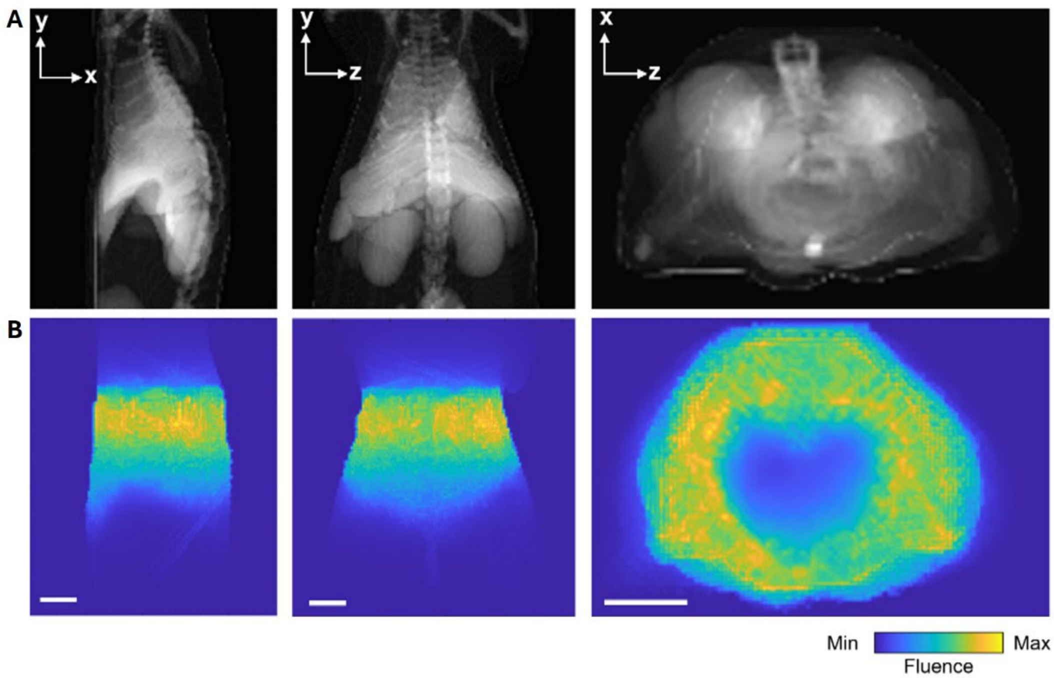

Fig S5. Simulation of light delivery for full-view PACT system. (A) MicroCT scan of the mouse model. (B) Optical fluence distribution from the Monte Carlo simulation. Scale bar: 5 mm.

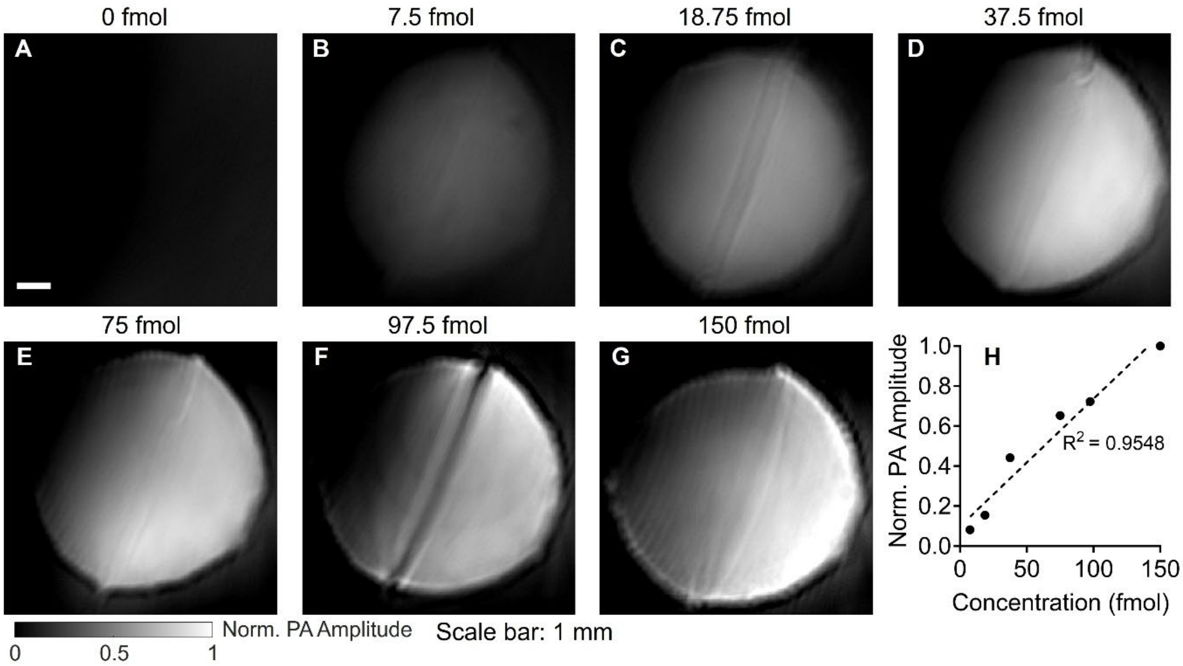

Fig S6. Relationship between AC-GNS concentration and PA signal amplitude. (A-G) PA images of AC-GNS-3 at increasing concentration (0-150 fmol). (H) Linear fitting between PA signal amplitude and concentration. Scale bar: 1 mm.

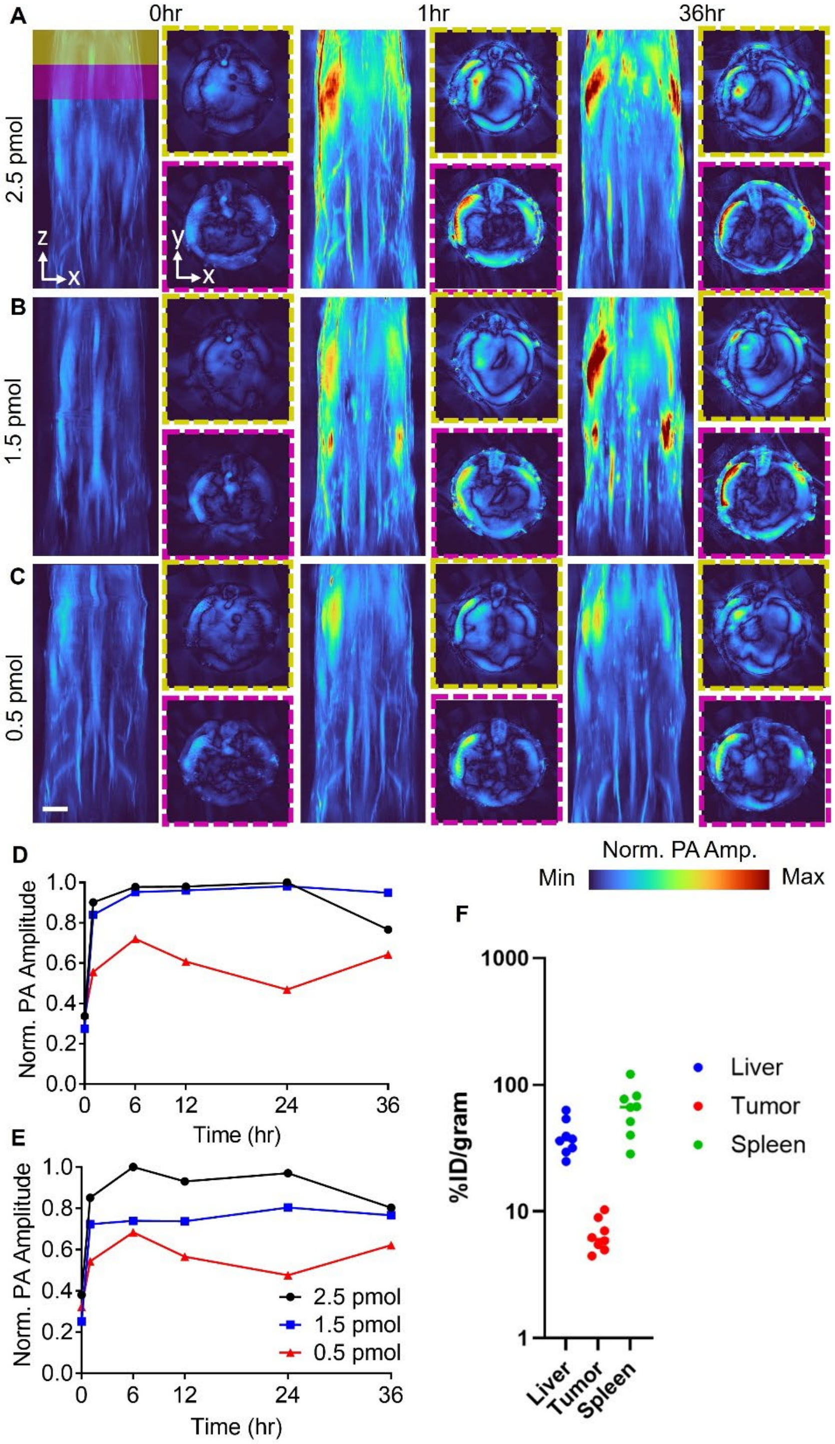

Fig S7. Whole-body AC-GNS biodistribution tracking with full-view PACT system. Biodistribution monitoring at liver (purple) and spleen (yellow) with (A) 2.5 pmol, (B) 1.5 pmol and (C) 0.5 pmol of AC-GNS respectively. Longitudinal average PA signal amplitude at (D) liver and (E) spleen, N=1. (F) ICP-MS analysis of liver, tumor and spleen of mice injected with 0.5 pmol of AC-GNS particles, N=8. Scale bar: 5 mm.

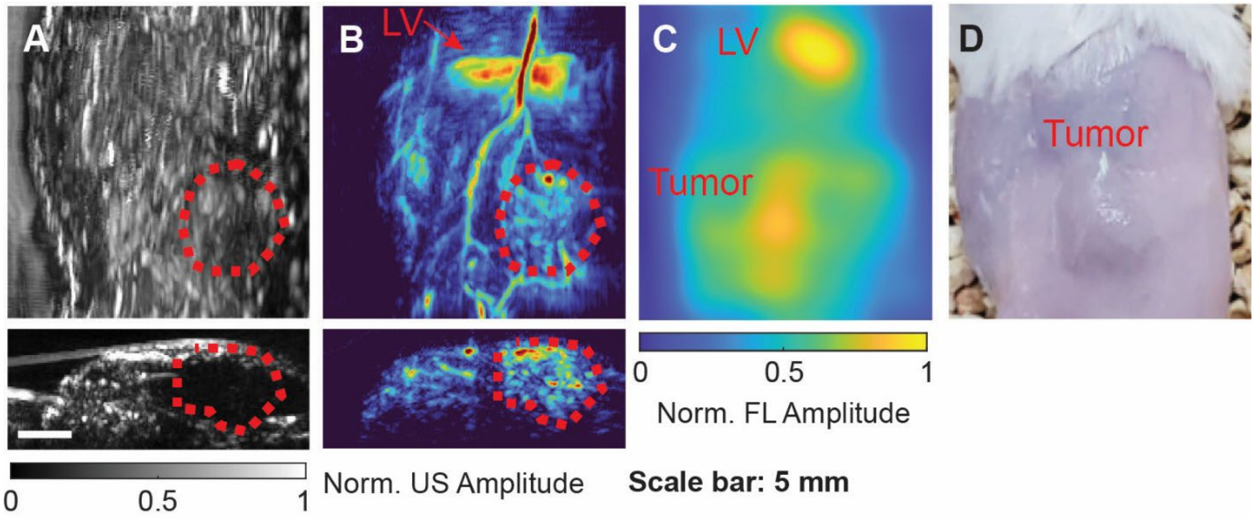

Fig S8. **Whole-body AC-GNS biodistribution tracking verification with linear-array PACT system and FL imaging.** (A) US, (B) linear-array PACT, (C) FL imaging and (D) photograph of the tumor and liver regions. The mouse was injected with 1.5-pmol RcGNS. LV, liver. Scale bar: 5mm.

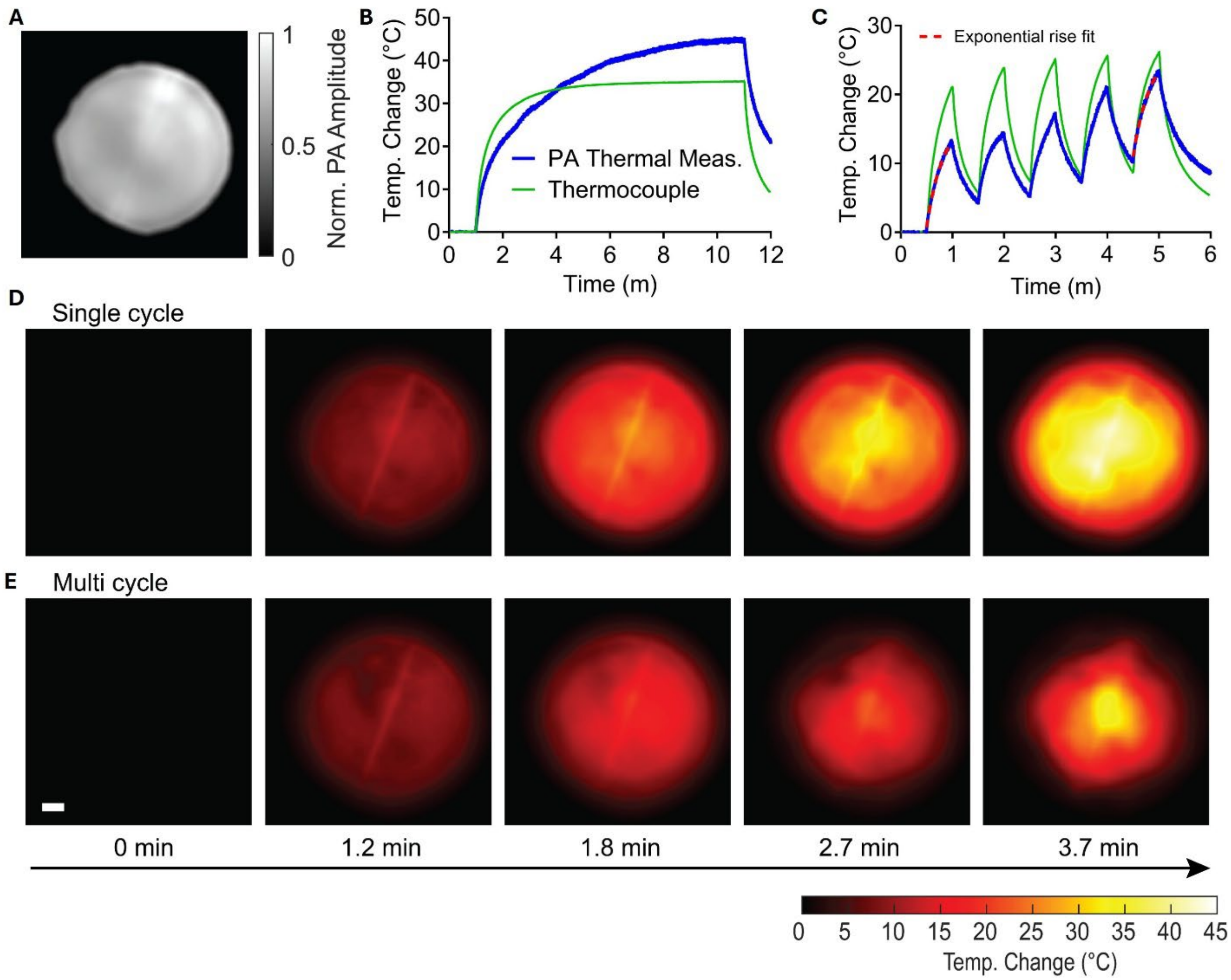

Fig S9. Validation of PA thermometry using thermocouple. (A) PA image of the tumor phantom (B) Single-cycle and (C) multi-cycle measurements from PA thermometry and thermocouple. Representative PA thermal maps of (D) single-cycle and (E) multi-cycle treatment at incremental timepoints. Scale bar: 1 mm.

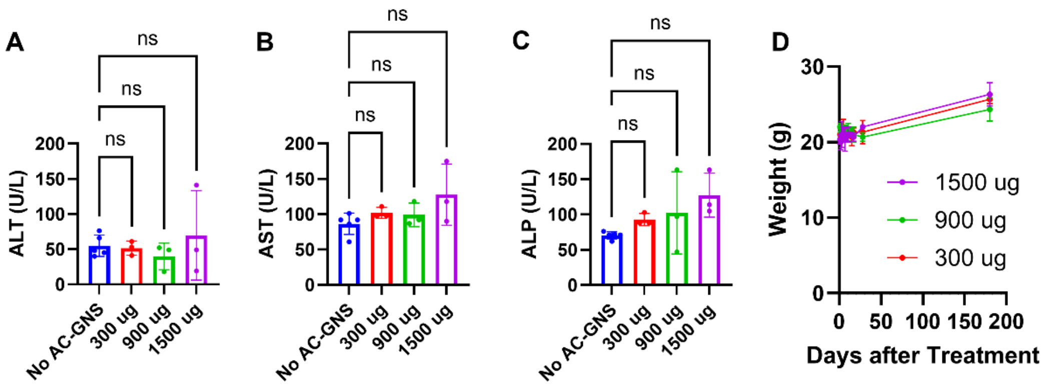

Fig S10- Long term Rc-GNS biocompatibility. (A) Circulating alanine aminotransferase levels of long-term survivors vs controls, N= 3 per group. (B) Circulating aspartate transferase levels of long-term survivors vs controls, N= 3 per group. (C) Circulating alkaline phosphatase levels of long-term survivors vs controls, N= 3 per group. (D) animal body weight per group after AC-GNS injection, N= 3 per group.

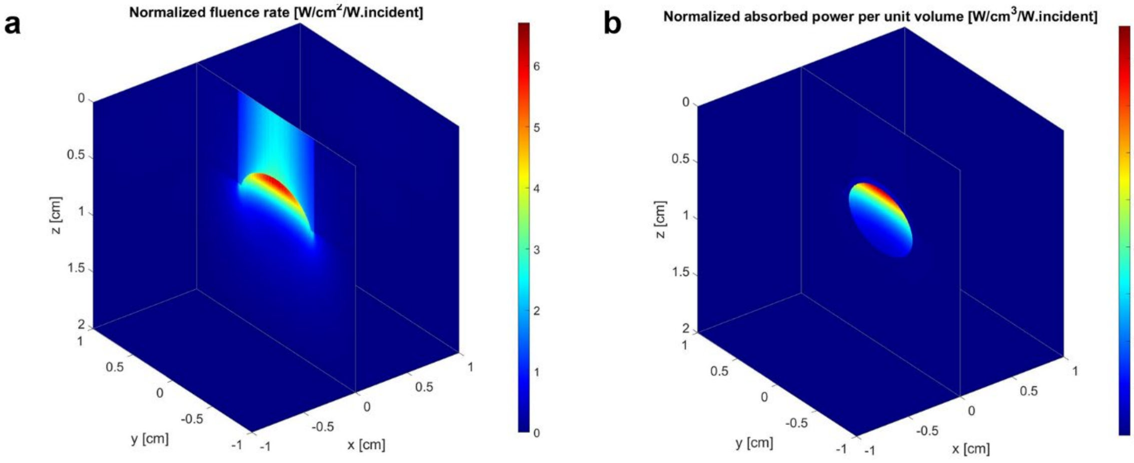

Fig S11. Simulated open-air and submerged PTT. A) Normalized fluence of treatment model determined using MCmatlab. B) Normalized absorbed power during treatment simulation.

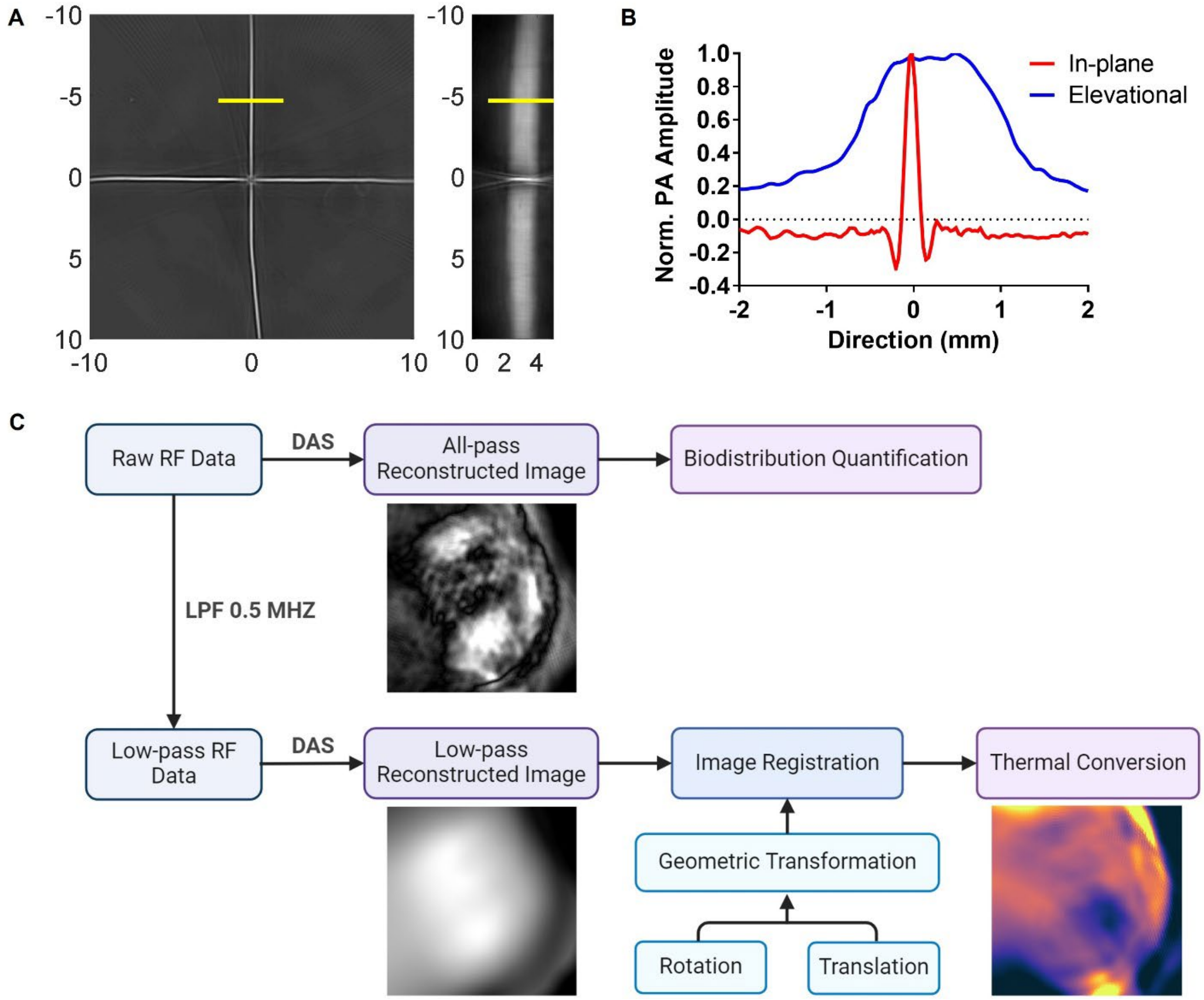

Fig S12. Quantification and signal processing of full-view PACT system. (A) PA images of the crossing hairs as resolution target. (B) Line profiles showing in-plane and elevational resolution. C) PA signal processing pipeline of the integrated full-view PACT/PTT system.

Movie S1.

Spatial EELS intensity as a function of energy across the entire spectrum image.

Movie S2.

In vivo real-time thermal mapping performed via PACT. Top Left- Bipolar image of the tumor region during photothermal treatment. Top Right- Thermal map of tumor region during photothermal treatment. Bottom Left- Merged view of bipolar PA image and thermal map of tumor region during photothermal treatment. Bottom Right- average temperature change across the tumor region during photothermal treatment.
